# Interplay between Energy and Entropy Mediates Ambimodal Selectivity of Cycloadditions

**DOI:** 10.1021/acs.jctc.4c01138

**Published:** 2024-12-06

**Authors:** Wook Shin, Yaning Hou, Xin Wang, Zhongyue J. Yang

**Affiliations:** † Department of Chemistry, Vanderbilt University, Nashville, Tennessee 37235, United States; ‡ Center for Structural Biology, Vanderbilt University, Nashville, Tennessee 37235, United States; § Vanderbilt Institute of Chemical Biology, Vanderbilt University, Nashville, Tennessee 37235, United States; ∥ Department of Chemical and Biomolecular Engineering, Vanderbilt University, Nashville, Tennessee 37235, United States; ⊥ Data Science Institute, Vanderbilt University, Nashville, Tennessee 37235, United States; # Henan-Macquarie University Joint Centre for Biomedical Innovation, School of Life Sciences, Henan University, Kaifeng, Henan 475004, China

## Abstract

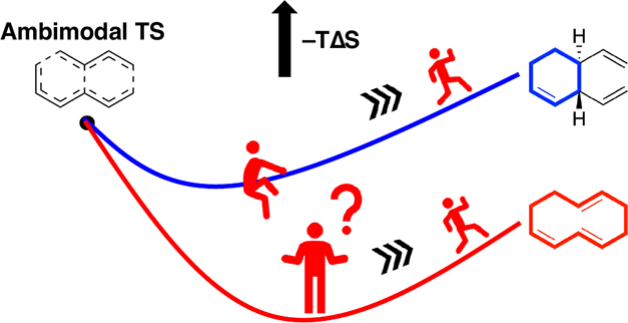

One ambimodal transition state can lead to the formation of multiple products. However, it remains fundamentally unknown how the energy and entropy along the post-TS pathways mediate ambimodal selectivity. Here, we investigated the energy and entropy profiles along the post-TS pathways in four [4 + 2]/[6 + 4] cycloadditions. We observe that the pathway leading to the minor product involves a more pronounced entropic trap. These entropic traps, resulting from the conformational change in the dynamic course of ring closure, act as a reservoir of longer-lived dynamic intermediates that roam on the potential energy surface and have a higher likelihood of redistributing to form the other product. The SpnF-catalyzed Diels–Alder reaction produces [4 + 2] and [6 + 4] adducts with nearly equal product distribution and relatively flat energy profiles, in contrast to other cycloadditions. Unexpectedly, the entropy profiles for these two adducts are distinctly different. The formation of the [6 + 4] adduct encounters an entropic barrier acting as a dynamical bottleneck, while the [4 + 2] adduct involves a substantial entropic trap to maintain long-lived intermediates. These opposing effects hinder both product formations and likely cancel each other out so that an equal product distribution is observed.

## Introduction

Post-transition-state bifurcation (PTSB) refers to a phenomenon in which one single transition state (TS), so-called ambimodal TS, leads to the formation of multiple products.^1−5^ The ambimodal selectivity caused by PTSBs has been observed in many chemical reactions ranging from pericyclic reactions,^6−17^ nucleophilic reactions,^18^ organometallic catalysis,^19^ to biosynthetic transformations.^20−23^ To investigate the impact of PTSB on product ratio, direct dynamics trajectory simulations have been extensively employed, from which ambimodal selectivity is predicted by counting the distribution of trajectories that fall into different product regions.^3^ Analytical models, statistical models,^24,25^ and machine learning models^26^ have been developed to predict ambimodal selectivity based on the features of the transition-state structure (TSS),^9,25,27^ valley ridge inflection point,^28^ or topology of the potential energy surface (PES).^29,30^

Nonetheless, our fundamental understanding of the ambimodal selectivity is incomplete. Current models predominantly focus on the impact of the initial state of PTSB (i.e., TS) on the product distribution, considering factors like TS geometry,^9^ momentum,^27^ and kinetic energy distribution.^25^ These TS-based models implicitly assume that the initial conditions dictate the outcome of reaction dynamics. This hypothesis likely breaks when trajectories are longer (e.g., >100 fs), the conformational distribution of reacting species broadens (e.g., entropic intermediates^31^), energy gradients between bifurcating pathways become similar, and multiple subsequent bifurcation occurs in the course of reaction dynamics simulations (e.g., trispericyclic cycloaddition^6^ and carbocation rearrangement^32^). One notable example is the dynamic study of carbocation rearrangement by Hare et al., in which energy gradients along the PTSB pathways were shown to mediate ambimodal selectivity.^29^ In these situations, apparent linear correlation between TS features and the product outcomes, even if it exists, does not reveal its dynamical complexity. The contributions of fleeting intermediates to ambimodal selectivity remain unknown. Although phase space models have been established for elucidating the dynamical basis of roaming and dynamic matching in simple polyatomic systems, full dimensional phase space analysis of complex molecules appears infeasible.^33−37^ To elucidate the full mechanistic picture of ambimodal selectivity, a comprehensive analysis into the energy and entropy profiles along the formation of each bifurcating product is essential.^38^

In this study, we investigated how the interplay between energy and entropy mediates ambimodal selectivity in PTSBs yielding [4 + 2] and [6 + 4] adducts using our previously developed bidirectional generative adversarial network–entropic path sampling (BGAN-EPS) method.^39^ Notably, different from equilibrium thermodynamic entropy, the calculated entropy profile was obtained using the formalism of configurational entropy, reflecting the changes in entropy due to variations in the geometric space of the trajectory ensemble at different stages of the PTSB.^39,40^ We evaluated post-TS energy (calculated by averaging productive trajectories) and entropy profiles in four cycloadditions, including the diene/triene cycloaddition, the tethered-diene/triene cycloaddition, the NgnD-catalyzed Diels–Alder reaction, and the SpnF-catalyzed Diels–Alder reaction. These studies complement existing TS-centric models and provide a more complete mechanistic picture of the origin of ambimodal selectivity.

## Results and Discussion

### Overview of the BGAN-EPS Method for Entropy Profile Computation

**Figure 1:**
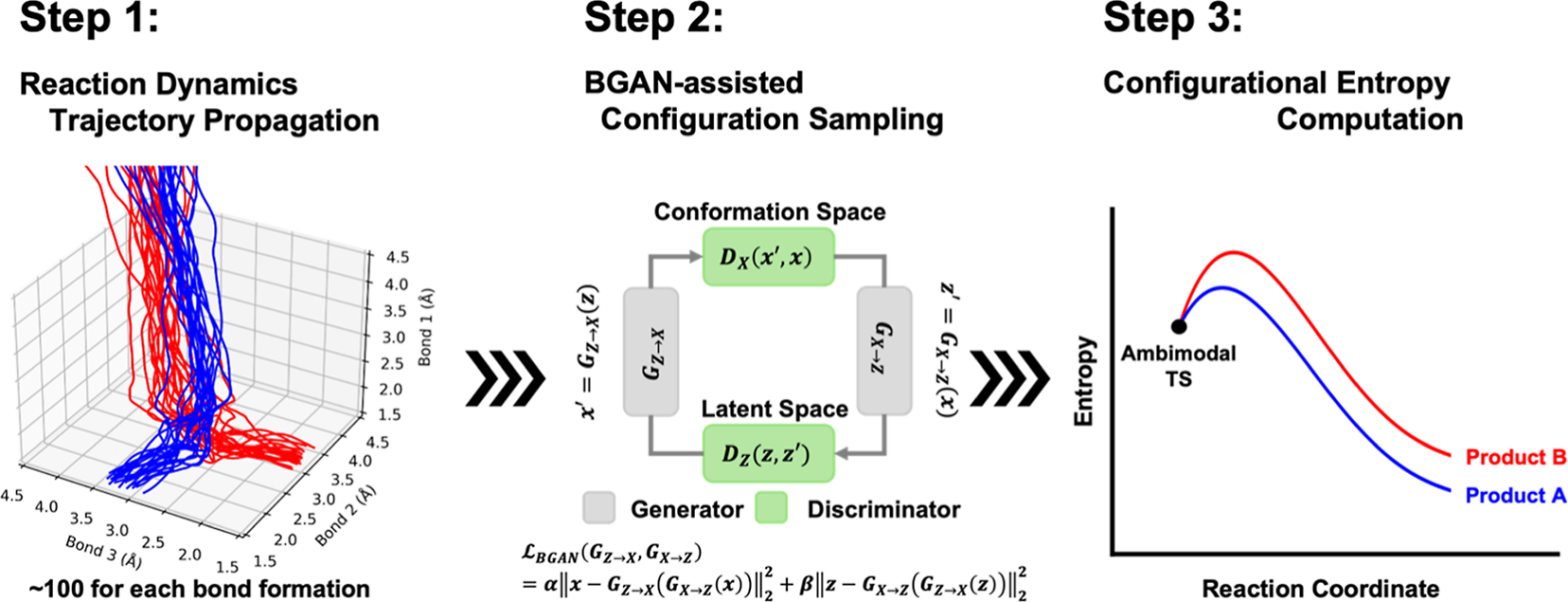
Overview of the BGAN—entropic path sampling method. Step 1 initiates reaction dynamics trajectories from the TSS, obtaining a minimum number of ca. 100 trajectories for each product formation. Step 2 involves training the BGAN model with molecular configurations sampled from step 1, which accelerates the evaluation of the probability density function of molecular configurations by generating statistically indistinguishable pseudomolecular configurations. Step 3 plots the configurational entropy profile along a user-defined reaction coordinate (i.e., reacting bond) using pseudomolecular configurations generated from step 2.

The BGAN-EPS method uses an optimized TSS as the input and generates an entropy profile along a user-defined reaction coordinate as the output. The method comprises three steps: (1) *reaction dynamics trajectory propagation*, (2) *BGAN-assisted configuration sampling*, and (3) *configurational entropy computation* (Figure 1).

Step 1 propagates the reaction dynamics trajectories. Initiated from the input TSS, we propagated quasiclassical trajectories (QCTs) in the gas phase using Progdyn, where the force and energy were calculated on the fly with the B3LYP-D3/6-31G(d) method in Gaussian16 (Supporting Information Table S1).^41−48^ To minimize statistical errors when comparing entropy profiles between the two bifurcating pathways and across different cycloadditions, we randomly selected a set of 102 QCTs each for the formation of [4 + 2] and [6 + 4] adducts in all cycloadditions.

Using input trajectories for each formed adduct, step 2 constructs a training set by converting Cartesian coordinates into redundant internal coordinates based on bonding connectivity. Subsequently, the BGAN model generates pseudomolecular configurations that are statistically indistinguishable from the training set configurations (Supporting Information Table S2), thereby enhancing the sampling of molecular configurations. Notably, the model shows a tendency to prefer generating pseudomolecular configurations near the TS region, underrepresenting the structural ensemble nearby the product forming region (i.e., below 2.0 Å).^39^ To mitigate the impact of this biased sampling problem on the generated entropic profile, the method excludes any generated structural ensembles that are less than 25% of the largest populated structural ensemble along the post-TS reaction pathway (Supporting Information Tables S5–S8).

Using the generated pseudomolecular configurations from the BGAN model, step 3 evaluates the entropy profile along a user-defined reaction coordinate (i.e., reacting bond) using entropic path sampling.^40^ The pseudomolecular configurations are segmented into multiple structural ensembles along the reaction coordinate (Supporting Information Table S3). Configurational entropy for each structural ensemble is calculated by adding the entropy for each of the 3*N*-6 vibrational degrees of freedom, including *N*-1 bonds, *N*-2 angles, and *N*-3 torsion angles (Supporting Information, Text S1). Higher-order entropy terms are computed using the maximum information spanning tree approximation.^49^ The resulting entropy profile is calculated relative to the first truncated ensemble near the TS.

### Model Systems of Ambimodal Cycloadditions

Using the BGAN-EPS method, we calculated the post-TS entropy profiles for the diene/triene cycloaddition, the tethered-diene/triene cycloaddition, the NgnD-catalyzed Diels–Alder reaction, and the SpnF-catalyzed Diels–Alder reaction. Our prior studies established the BGAN-EPS method using symmetric PTSBs that form an identical product via different bonding patterns.^39^ In contrast, the four model reactions used here yield different types of products via asynchronous bond formation. To assess the robustness of the BGAN-EPS method, we generated 10 entropy profiles for each bifurcating product in the diene/triene cycloaddition reaction, in which each entropy profile was computed using a distinct set of 102 trajectories (Supporting Information Figure S1). The calculated profiles exhibit highly consistent trends across all sets, demonstrating the reliability of the BGAN-EPS method in capturing entropy variations.

**Figure 2:**
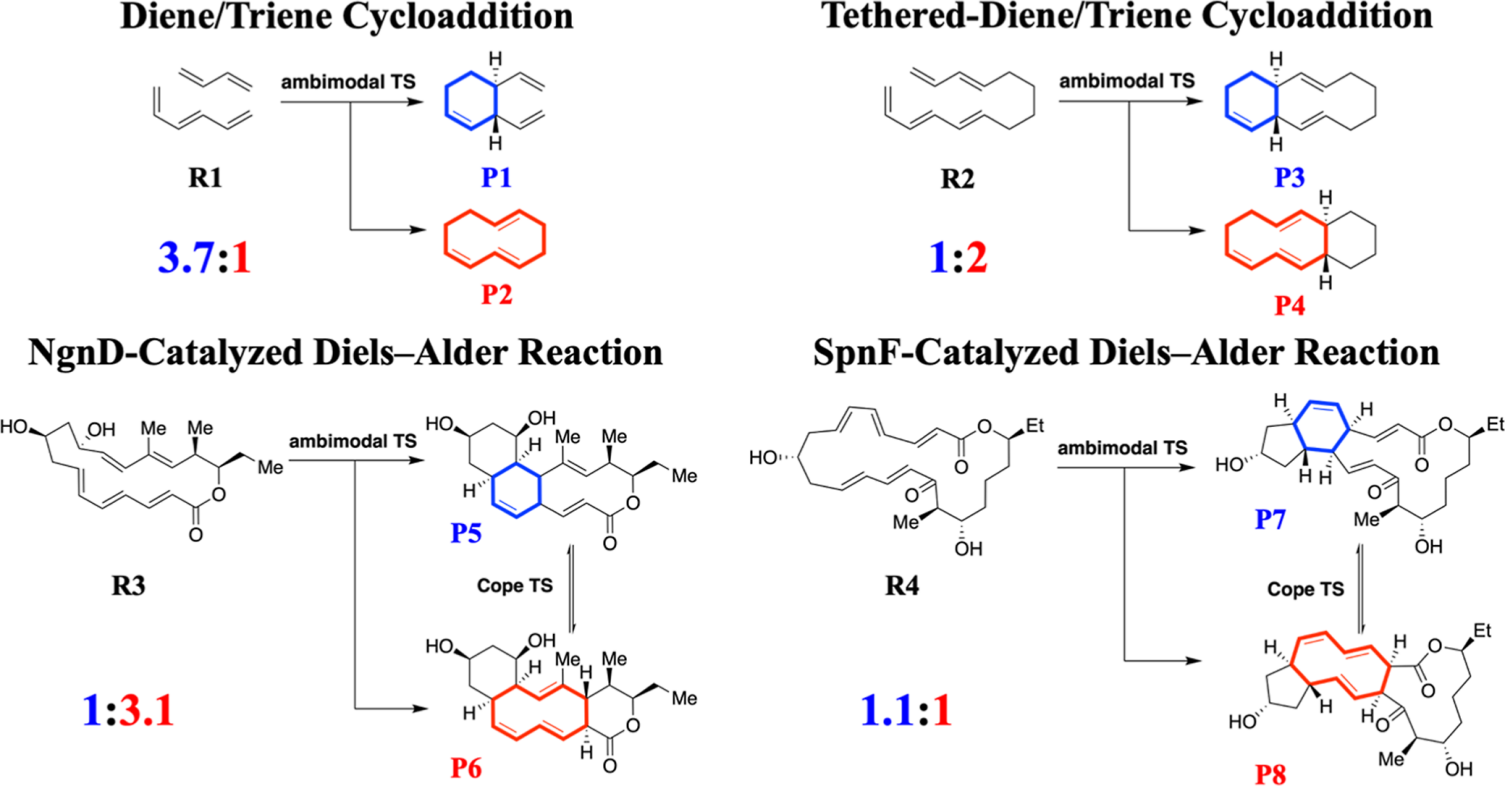
Reaction schemes and product ratios of the diene/triene cycloaddition, the tethered-diene/triene cycloaddition, the NgnD-catalyzed Diels–Alder reaction, and the SpnF-catalyzed Diels–Alder reaction in the gas phase. Blue represents the [4 + 2] adduct and red represents the [6 + 4] adduct.

The respective product ratios of [4 + 2] (blue) to [6 + 4] adducts (red) are 3.7:1, 1:2, 1:3.1, and 1.1:1 in the gas phase (Figure 2). The diene/triene cycloaddition and the tethered-diene/triene cycloaddition are ambimodal pericyclic reactions involving butadiene with hexatriene and its tethered transannular counterpart, respectively. In this study, these two reactions will serve as model systems for investigating the intrinsic entropic behavior underlying the selectivity of [4 + 2]/[6 + 4] bifurcation. The NgnD-catalyzed Diels–Alder reaction is the first natural enzyme-catalyzed [6 + 4] cycloaddition in the biosynthesis of streptoseomycin, in which both [4 + 2] and [6 + 4] adducts were identified experimentally.^50,51^ Similarly, the SpnF-catalyzed Diels–Alder reaction is the first natural enzyme-catalyzed [4 + 2] cycloaddition in the biosynthesis of spinosyn A.^52^

In the following sections, we will demonstrate how entropy influences the [4 + 2]/[6 + 4] bifurcation selectivity by computing an entropy profile for each formed adduct using the BGAN-EPS method. We also calculated energy profiles by averaging the electronic energies of conformations within each structural ensemble along post-TS reaction pathways (see the Computational Methods section for more details). The energy profiles that lead to the formation of the major product align closely with the intrinsic reaction coordinate (IRC) (Supporting Information Figure S2). For each reaction, we assessed the interplay between the entropy and energy along the post-TS reaction pathways. Furthermore, we investigated how local structural moieties, such as the 4π, 6π, propyl or butyl tether, and ester tether moieties, contribute to the difference in entropy profiles of the two bifurcating adducts. To ensure a consistent comparison between the asynchronous bond formation processes, the *x* axes of both energy and entropy profiles are shown in change of forming bond lengths with reference to those in the TSSs (i.e., bond 2 and bond 3 for the [4 + 2] and [6 + 4] adduct, respectively).

### Diene/Triene Cycloaddition

**Figure 3:**
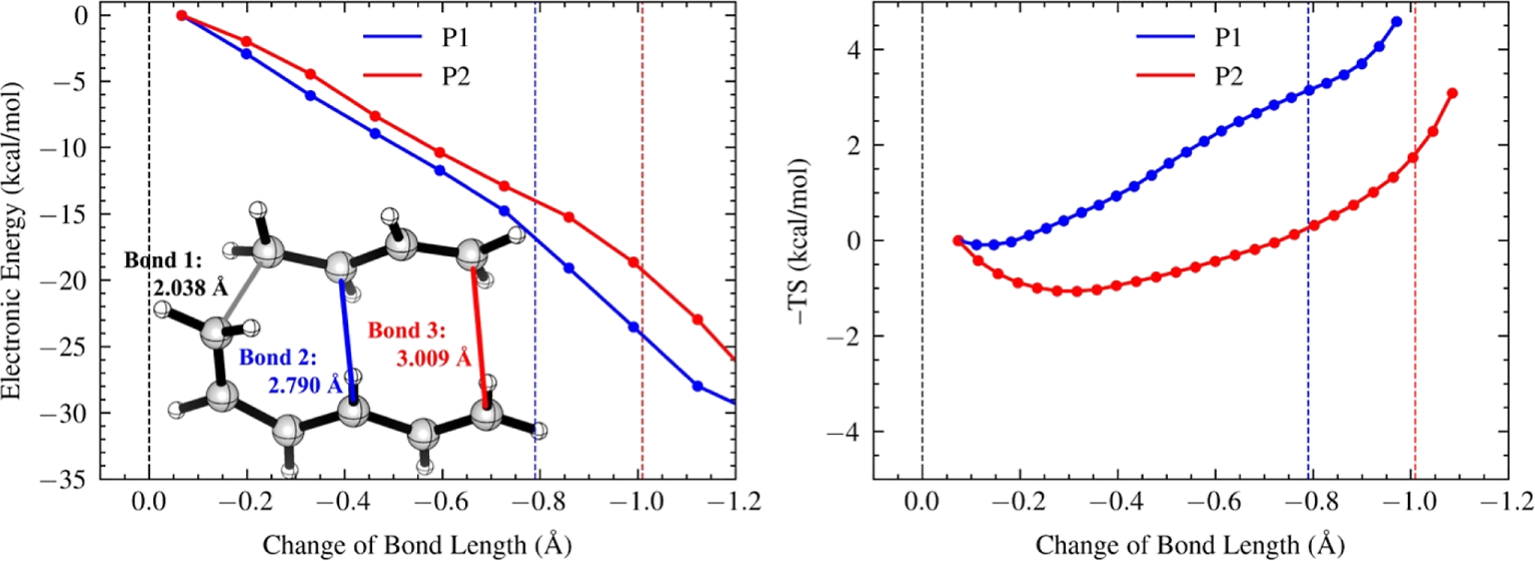
Energy (left) and entropy (right) profiles, alongside the ambimodal TSS of the diene/triene cycloaddition. Bond 2 formation leads to the [4 + 2] adduct P1 (blue), and bond 3 formation leads to the [6 + 4] adduct P2 (red). The *x*-axes show changes in bond lengths from the ambimodal TSS, with blue and red dashed lines indicating 2.0 Å. The energy and entropy values for each structural window are computed with reference to those of the first post-TS points. Error bars on energy and entropy profiles are invisible due to its small scale (Supporting Information Table S9).

In the diene/triene cycloaddition, the formation of [4 + 2] adduct (P1) is observed as the major product, while the [6 + 4] adduct (P2) is the minor product (*upper left*, Figure 2). Along the post-TS reaction pathway on the PES, P1 follows a steeper energy gradient downhill than P2. Besides differences in the energy gradients, we observed distinct entropy profiles along their respective post-TS reaction paths (*right*, Figure 3). Both the formation of P1 and P2 involve entropic traps, which act as a reservoir for long-lived dynamic intermediates with enhanced conformational flexibility compared to the ambimodal TS. Specifically, P2 encounters a deeper entropic trap with a −*T*Δ*S* value of −1.06 kcal/mol at −0.30 Å, while P1 encounters a shallow entropic trap with a −*T*Δ*S* value of −0.09 kcal/mol at −0.13 Å.

To elucidate the dynamic basis underlying the formation of an entropic trap, we analyzed the composition of roaming dynamic species at each stage of the post-TS pathway (Supporting Information Figure S3). These roaming species are defined here as those exhibiting a near-zero velocity, specifically within the range of −0.01 to 0.01 Å/fs. We observed that P2 consistently exhibits a higher population of roaming species (by about 20%) than P1 until the later stage of the reaction near product formation. The higher population of roaming species contributes to the formation of a deeper entropic trap in P2. These roaming species, though initially heading toward P2, bear the potential of rerouting to the other bifurcating pathway and ultimately form P1 (see an example from Supporting Information Figure S4). In contrast, the shallower entropic trap for P1 is associated with a smaller population of roaming species, promoting direct formation of the final product. Notably, although the energy reduction from the TS spans over 30 kcal/mol across the entire post-TS pathway, the relative energy difference between P1 and P2 at each stage is only about 2 kcal/mol, which is comparable to the difference in entropy profiles. These indicate that P1’s favorable ambimodal selectivity is associated with its steeper energy gradient and shallower entropic trap.

To investigate the origin of different entropic traps between P1 and P2, we decomposed molecular configurational entropies into contributions of local structural moieties (Supporting Information Figure S5). For P1, the entropy profiles of both 4π and 6π moieties exhibit a similar trend with a shallow entropic trap with −*T*Δ*S* values of −0.06 and −0.03 kcal/mol, respectively. In contrast, the 4π and 6π moieties in P2 show more pronounced entropic traps with −*T*Δ*S* values of −0.58 and −0.50 kcal/mol, respectively. The formation of the [4 + 2] adduct (P1) necessitates the alignment of the 2π (a component of 4π) and 4π (a component of 6π) moieties to form a 6-membered ring, which is more conformationally strained than a 10-membered ring of the [6 + 4] adduct (P2). As such, P2 shows greater conformational flexibility in the 4π and 6π moieties, resulting in a deeper entropic trap.

### Tethered-Diene/Triene Cycloaddition

**Figure 4:**
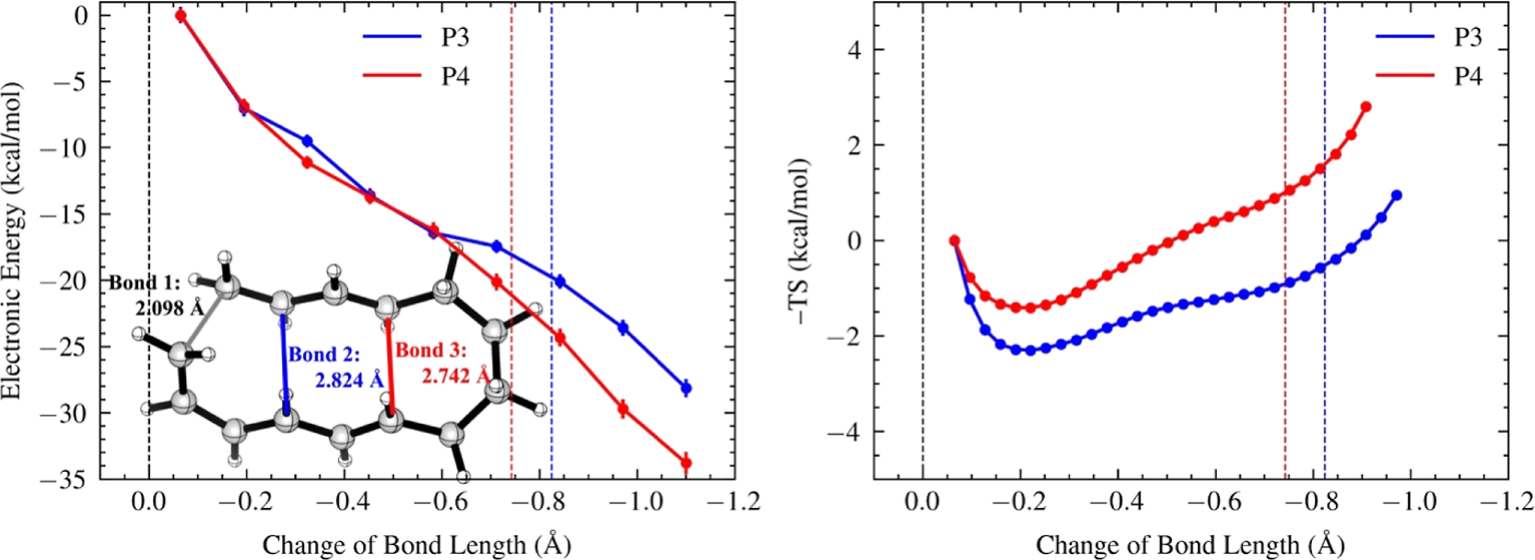
Energy (left) and entropy (right) profiles, alongside the ambimodal TSS of the tethered-diene/triene cycloaddition. Bond 2 formation leads to the [4 + 2] adduct P3 (blue), and bond 3 formation leads to the [6 + 4] adduct P4 (red). The *x*-axes show changes in bond lengths from the ambimodal TSS, with blue and red dashed lines indicating 2.0 Å. The energy and entropy values for each structural window are computed with reference to those of the first post-TS points. Error bars on energy and entropy profiles represent the standard error of the mean.

In the tethered-diene/triene cycloaddition, one side of the terminal alkenes of butadiene and hexatriene is connected by a butyl linker (Figure 4). The formation of the [6 + 4] adduct (P4) is more favorable than the [4 + 2] adduct (P3), demonstrating an opposite trend to what is observed in the diene/triene cycloaddition (*upper right*, Figure 2). Unlike the untethered [4 + 2]/[6 + 4] bifurcation, where P1 and P2 trajectories exhibit different energetic steepness in their descent down the PES from the ambimodal TS, P3 and P4 trajectories in the tethered system display similar energetic downhill trends up to −0.6 Å (*left*, Figure 4). This indicates that the energy gradient in the vicinity of the TS does not have preference over either product in the initial stage of the post-TS pathway. However, within this region, P3 and P4 trajectories encounter different depths of entropic traps (*right*, Figure 4). The P3 trajectories, which lead to the minor product, encounter a deeper entropic trap of −2.30 kcal/mol at −0.22 Å, while the P4 trajectories, which lead to the major product, experience a shallower trap of −1.41 kcal/mol at −0.21 Å. Similar to the diene/triene cycloaddition, we elucidated the dynamic basis of the entropic traps for P3/P4 by analyzing the population of roaming dynamic species at each stage of the post-TS pathway (Supporting Information Figure S3). Within the entropic trap region (i.e., from −0.2 to −0.4 Å), P3 involves a more prevalent population of roaming species than P4, contributing to a higher likelihood for trajectory rerouting that makes the [6 + 4] adduct the major product. This demonstrates that in the early stage of the post-TS pathway when energy gradients are nearly identical, entropy arising from dynamic roaming becomes the key factor in determining selectivity.

To elucidate why the [4 + 2]-adduct (P3)-forming trajectories involve a deeper entropic trap, we decomposed the entropy profiles in the tethered-diene/triene cycloaddition into contributions from the 4π, 6π, and butyl tether moieties (Supporting Information Figure S6). The 4π, 6π, and butyl tether moieties in P3 show deeper entropic traps than those in P4 by 0.26, 0.33, and 0.26 kcal/mol, respectively. Opposite to the trend observed in P1 versus P2, the 4π and 6π moieties in the [4 + 2] adduct (P3) show deeper entropic traps than those in the [6 + 4] adduct (P4). Compared to P2, the formation of P4 strains the alkyl chain to a six-membered ring with the butyl tether, narrowing the conformational distribution of P4’s 4π and 6π moieties, reducing their entropy contributions by 0.36 and 0.17 kcal/mol, respectively. In contrast, compared to P1, the formation of P3 leads to a 10-membered ring with broader conformational distribution, thereby increasing the entropy contribution of P3′s 4π and 6π moieties by 0.42 and 0.63 kcal/mol, respectively. Although both adducts will end up with a polycyclic ring with two adjacent 6-membered/10-membered rings, the conformational straining with the butyl tether helps the P4-forming trajectories reduce the lingering time in the entropic trap. Notably, the conformational straining, which refers to the narrowing of the conformational distribution accompanying dynamic bond formation, is different from the concept of “ring strain” broadly discussed in physical organic chemistry. The latter emphasizes the enthalpic instability of ring formation due to more C–H repulsion.

### NgnD-Catalyzed Diels–Alder Reaction

**Figure 5:**
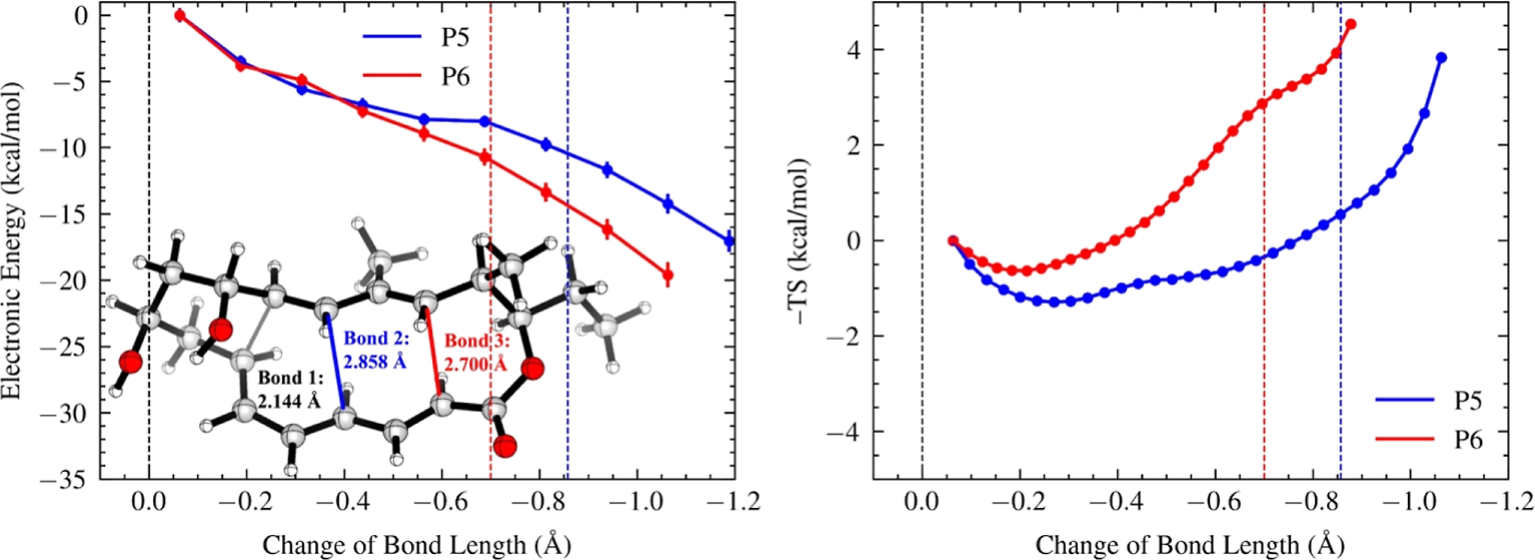
Energy (left) and entropy (right) profiles, alongside the ambimodal TSS of the NgnD-catalyzed Diels–Alder reaction. Bond 2 formation leads to the [4 + 2] adduct P5 (blue), and bond 3 formation leads to the [6 + 4] adduct P6 (red). The *x*-axes show changes in bond lengths from the ambimodal TSS, with blue and red dashed lines indicating 2.0 Å. The energy and entropy values for each structural window are computed with reference to those of the first post-TS points. Error bars on energy and entropy profiles represent the standard error of the mean.

Different from the tethered-diene/triene cycloaddition, the substrate of the NgnD-catalyzed Diels–Alder reaction (R3) is extensively functionalized. The butyl linker is replaced with an ester moiety (ethyl formate), and the terminal alkenes are substituted with a dihydroxy-butyl linker, which forms a local 6-membered ring upon the formation of bond 1 in the products. Additionally, alkyl groups are added to multiple carbons of the scaffold. Despite these significant structural modifications, the dynamic and thermodynamic behaviors of the NgnD-catalyzed Diels–Alder reaction closely mirror those observed in the tethered-diene/triene cycloaddition. The [6 + 4] adduct (P6) continues to be the major product, while the [4 + 2] adduct (P5) remains the minor product (*lower left*, Figure 2). Similar energy gradients are observed between P5 and P6 up to −0.6 Å (*left*, Figure 5), but the entropy profiles are substantially different. The major product, P6, encounters a shallow entropic trap (−*T*Δ*S* = −0.63 kcal/mol at −0.20 Å) before its entropy profile heads straight up, corresponding to a direct product formation without much dynamic roaming. However, the minor product P5 involves a significantly wider entropic region, with its population of roaming species being 40% higher than that of P6 even beyond −0.6 Å.

The question is, why does the structural modification in the NgnD-catalyzed Diels–Alder reaction not affect the entropic trend from the tethered diene/triene cycloaddition in the early stage of the post-TS pathway? To address this, we partitioned the entropy contributions to four local structural moieties, including the 4π, 6π, ester tether, and butyl tether moieties (Supporting Information Figure S7). The entropic traps associated with P5′s 4π, 6π, ester tether, and butyl tether moieties are deeper than those in P6 by 0.08, 0.41, 0.35, and 0.11 kcal/mol, respectively. This pattern can also be explained by the entropic preferences associated with conformational straining during bond formation: the 10-membered ester ring formed during the P5 trajectories is more conformationally flexible than the 6-membered ester ring formed in the P6 trajectories. This trend remains unaffected by alkyl functionalization in the 4π and ester tether moieties. In addition, the butyl tether’s impact on differentiating entropic traps of both bifurcating pathways appears minimal. This is likely because the butyl tether contributes to the formation of bond 1, which is shared by both products. In contrast, the ester tether, located near bonds 2 and 3, has a more substantial effect on ambimodal selectivity. As such, the influence of structural modifications on the entropy appears to be localized.

### SpnF-Catalyzed Diels–Alder Reaction

Compared to the NgnD-catalyzed Diels–Alder reaction, the substrate of the SpnF-catalyzed Diels–Alder reaction also features an alkyl linker near bond 1 and an ester tether near bond 3 but with a different configuration. The alkyl tether is replaced with a hydroxypropyl linker, and the ethyl formate tether is replaced with a heptyl formate tether. These modifications result in the formation of a local five-membered ring with the propyl linker as bond 1 forms and, subsequently, 15-membered and 11-membered rings with the ester linker as bond 2 and bond 3 form, respectively.

**Figure 6:**
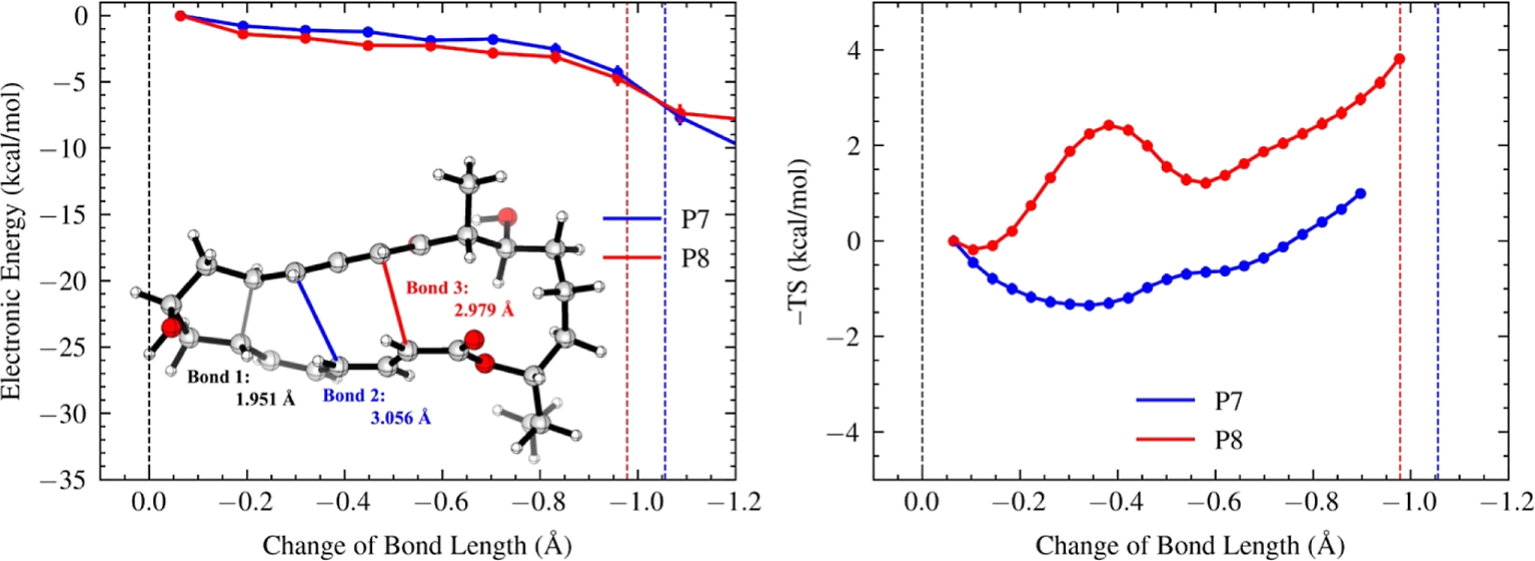
Energy (left) and entropy (right) profiles, alongside the ambimodal TSS of the SpnF-catalyzed Diels–Alder reaction. Bond 2 formation leads to the [4 + 2] adduct P7 (blue), and bond 3 formation leads to the [6 + 4] adduct P8 (red). The *x*-axes show changes in bond lengths from the ambimodal TSS, with blue and red dashed lines indicating 2.0 Å. The energy and entropy values for each structural window are computed with reference to those of the first post-TS points. Error bars on energy and entropy profiles represent the standard error of the mean.

The SpnF-catalyzed Diels–Alder reaction yields a nearly equal ratio between the [4 + 2] adduct (P7) and the [6 + 4] adduct (P8) (*lower right*, Figure 2). Regarding energetics, both P7 and P8 follow a flatter downhill PES than other reactions (R1–R3), though P8 is slightly steeper (*left*, Figure 6). Unexpectedly, P7 and P8 present remarkably distinct entropy profiles (*right*, Figure 6). The formation of P7 encounters an entropic trap at 0.34 Å with a −*T*Δ*S* value of −1.35 kcal/mol, indicative of long-lived dynamic intermediates. In contrast, the formation of P8 encounters an entropic barrier at 0.38 Å with a −*T*Δ*S* value of 2.42 kcal/mol, presenting a hurdle to overcome the dynamic bottleneck. Both entropic trap and entropic barrier serve to hold back the reaction dynamics from proceeding along the pathway of product formation, causing a similarly high tendency for roaming behavior up until a later stage of the post-TS pathway (80% roaming even at 0.6 Å, Supporting Information Figure S3). The entropic barrier, which was not observed in other cycloadditions (R1–R3), adds additional free energy cost for the formation of P8, offsetting its slight advantage for trending downhill along the PES. Notably, the entropic barrier aligns with the variational TS that occurs in the post-saddle point region as reported by Patel et al.,^21^ causing them to roam near the ambimodal TS region and recrossing (Supporting Information Figure S9). This also explains the high percentage of recrossing trajectories observed for the SpnF-catalyzed Diels–Alder reaction (i.e., 36%). This reaction exemplifies how the trade-off between energy and entropy factors mediates reaction dynamics, resulting in an equal distribution of product formation.

To understand the structural basis for P8 to engender a substantial entropic barrier, we investigated entropy contributions from the 4π, 6π, propyl tether, and ester tether moieties (Supporting Information Figure S8). In the formation of P7, the 4π, 6π, propyl tether, and ester tether moieties encounter entropic traps (i.e., −0.16 kcal/mol, −0.46 kcal/mol, −0.07 kcal/mol, and −0.70 kcal/mol, respectively). In P8, 4π, 6π, and the ester tether moieties encounter entropic barriers with −*T*Δ*S* values of 0.37, 0.33, and 1.44 kcal/mol above their respective entropic traps. The propyl tether does not involve an entropic barrier. Compared with P7, the ester tether in P8 increases the entropy cost by 2.14 kcal/mol, which is the most significant increase observed among all moieties. This substantial increase in entropy cost is due to the formation of a strained 11-membered ester ring in P8 relative to a more flexible 15-membered ester ring in P7. Similar to the butyl tether in the NgnD-catalyzed Diels–Alder reaction, the propyl tether in this reaction plays a minimal role in influencing ambimodal selectivity because it quickly forms a 5-membered ring in the early stage of PTSB. These results show how the ester tether shapes the entropic landscapes of PTSB trajectories through forming different sizes of rings with forming bonds, thereby mediating ambimodal selectivity.

### Comparison between VTST and BGAN-EPS

Finally, we compared the BGAN-EPS method with the VTST approach for calculating the entropy profiles. For this comparison, we selected the diene/triene cycloaddition and the SpnF-catalyzed Diels–Alder reaction, focusing on whether the VTST method could detect the subtle entropic trap in P1 and the entropic barrier in P8 (Supporting Information Figure S10). For VTST calculations, we applied Truhlar’s quasiharmonic corrections^53^ by raising vibrational frequencies below 100 cm^–1^ to this threshold by using the GoodVibes^54^ software developed by the Paton group. To ensure the consistency across frames, Truhlar’s method was applied to a uniform set of vibrational modes across frames along the reaction path.

The VTST-derived entropy profile for P1 exhibits a gradual decrease, followed by a relatively flat region, failing to capture the entropic trap identified in the BGAN-EPS-derived profile. For P8, while the VTST-derived profile indicates an entropic barrier with a −*T*Δ*S* value of 0.26 kcal/mol, this value is significantly lower than the barrier observed with BGAN-EPS (−*T*Δ*S* of 2.42 kcal/mol). This discrepancy arises from VTST’s limitation in accurately capturing the entropic contribution of low-frequency modes within the harmonic approximation framework. Furthermore, evaluating the entropy profile of the minor product using VTST requires the manual definition of a reaction coordinate, introducing potential arbitrariness. This added complexity, combined with its limitations in quantitative accuracy, demonstrates another drawback of VTST to evaluate entropic profiles for bifurcating paths.

These findings highlight the distinct advantages of the BGAN-EPS method. Unlike VTST, BGAN-EPS calculates entropy profiles based on reactive trajectories for both major and minor products, employing a histogram-based configurational entropy method to more effectively capture the change in entropy along the post-TS pathway. This approach includes the contribution from the torsional motions that are anharmonic and low frequency in nature. Overall, these results underscore the enhanced ability of BGAN-EPS to capture detailed entropy profiles and complex dynamic features along bifurcating reaction pathways, offering a more nuanced understanding of the entropic influences on reaction selectivity.

## Conclusions

Our work elucidates how the energy and entropy profiles along the PTSB pathways influence ambimodal selectivity in [4 + 2]/[6 + 4] cycloadditions, complementing existing models that rationalize the origin of ambimodal selectivity merely based on the TS. In the diene/triene cycloaddition, the major product (P1) is formed due to its steeper energy gradient and shallower entropic trap. However, the tethered-diene/triene cycloaddition and the NgnD-catalyzed Diels–Alder reaction show almost identical energy gradients near the TS region, making the depth of the entropic trap a critical factor for mediating ambimodal selectivity in these two reactions. Unexpectedly, the SpnF-catalyzed Diels–Alder reaction, despite having a nearly identical product distribution, features distinct entropy profiles. The formation of the [4 + 2] adduct involves an entropic trap, in which entrapped intermediates roam and reroute to the alternative product, while the formation of the [6 + 4] adduct involves an entropic barrier that increases free energy cost. The offset between the two factors leads to a nearly equal distribution of the two adducts. Notably, the BGAN-EPS method pinpoints this entropic barrier during the formation of the [6 + 4] adduct, informing the specific PTSB pathway that encounters the dynamical bottleneck. The level of mechanic complexity and resolution obtained from the entropic/energetic analyses of PTSB pathways goes beyond what can be offered by the variational TS theory^21^ or simple TS-based correlation analysis.^24^

## Computational Methods

### Quasiclassical Trajectory Simulation

Quasiclassical trajectory simulations were conducted in the gas phase at 298.15 K by using Singleton’s Progdyn program and the B3LYP-D3/6-31G(d) method in Gaussian 16.^41−48^ Trajectories were initiated in the vicinity of the TS using the normal-mode sampling method. Each real normal vibrational mode in the TSS was energized with zero-point energy and thermal energy. A set of geometries and velocities were then randomly sampled according to the Boltzmann distribution. Subsequently, the QCTs were propagated both forward and backward with a velocity-Verlet algorithm with a 1 fs integration step until either the product (bond formation criterion: 1.700 Å) or reactant formed. For the diene/triene cycloaddition, the simulation results show 7377 (74%) trajectories forming the [4 + 2] adduct, 1961 (20%) forming the [6 + 4] adduct, and 583 (6%) are recrossing. For the tethered-diene/triene cycloaddition, the simulation results show 102 (33%) trajectories forming [4 + 2] adduct, 197 (63%) forming [6 + 4] adduct, and 12 (4%) are recrossing. For the NgnD-catalyzed Diels–Alder reaction, the simulation results show 117 (19%) trajectories forming the [4 + 2] adduct, 370 (59%) forming the [6 + 4] adduct, and 141 (22%) are recrossing. For the SpnF-catalyzed Diels–Alder reaction, the simulation results show 132 (33%) trajectories forming the [4 + 2] adduct, 121 (30%) forming the [6 + 4] adduct, and 144 (36%) are recrossing.

### Trajectory-Derived Energy Profile

Using sampled QCTs, we computed energy profiles by averaging the electronic energies across conformations within each structural ensemble defined in step 3 of the BGAN-EPS protocol (Supporting Information Tables S17–S20). For each reaction, we adopted an equal number of trajectories for energetic averaging: 1961 for the diene/triene cycloaddition, 102 for the tethered-diene/triene cycloaddition, 117 for the NgnD-catalyzed Diels–Alder reaction, and 121 for the SpnF-catalyzed Diels–Alder reaction, with the number determined by that of the minor product. The trajectories from the major product collection are randomly selected. Along the reaction coordinate, we maintained consistency in the number and width of structural ensembles used for both energy and entropy profiling (Supporting Information Table S3). Typically, the initial stages of QCTs (i.e., in the vicinity of the TS) exhibit lower energies compared to the subsequent stages of the reaction pathway. To ensure a more accurate representation of the energy profile and mitigate the influence of added zero-point energies, we excluded the first three time steps from the initial trajectories.

### Variational TS Theory Calculation

Starting from the optimized TSS, an IRC calculation was performed using the B3LYP-D3/6-31g(d) method in Gaussian 16,^42−48^ with a step size of 0.01 Bohr and an ultrafine integration grid. Projected vibrational frequencies perpendicular to the reaction path were calculated for points along the mass-weighted reaction path. To generate VTST-derived entropy profiles with minimized impact from the small frequencies, Truhlar’s quasiharmonic corrections^53^ were applied by raising vibrational frequencies below 100 cm^–1^ to this threshold by using the GoodVibes^54^ software developed by the Paton group. To ensure the consistency across frames, Truhlar’s method was applied to a uniform set of vibrational modes across frames along the reaction path.

### Data Preprocessing

Cartesian coordinates of molecular snapshots sampled from QCTs were converted into redundant internal coordinates based on bonding connectivity, thereby eliminating external rototranslational degrees of freedom. Using redundant internal coordinates minimizes information loss when representing molecular configurations. To mitigate statistical bias in model training, internal coordinates are independently normalized to a range between −1 and 1, based on their respective minimum and maximum values. These normalized internal coordinates were then structured into a two-dimensional array, serving as the BGAN training set for generating pseudomolecular configurations.

### Bidirectional Generative Adversarial Network

The BGAN model was developed to accelerate the entropic path sampling method by enhancing the sampling of molecular configurations.^39^ The objective of the BGAN model is to learn the probability distribution of molecular configurations (i.e., internal coordinates) sampled from QCTs and to generate pseudomolecular configurations that are statistically indistinguishable from those observed in the original molecular configurations.

The BGAN model comprises two pairs of generators and discriminators. In the model, coordinate variable ***x*** and latent variable **z** are treated as independent and identically distributed random variables with respective probability densities **p**(**x**) and **p**(**z**) in coordinate space **X** and latent space **Z**.  generates pseudomolecular configurations  that are statistically indistinguishable from the molecular configurations sampled from QCTs **x**. The discriminator **D**_**X**_ serves as a binary classifier distinguishing the generated data  from the real data (**D**_**X**_(**x**) = 1). Similarly,  transforms the original molecular configurations **x** into pseudolatent variable , and **D**_**Z**_ differentiates generated data  from the real data (**D**_**Z**_(**z**) = 1).

The objective loss functions for these four neural networks (i.e., , and **D**_**Z**_) are defined as follows during the training process
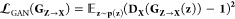
1
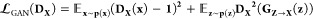
2
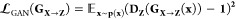
3
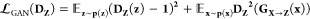
4

During the joint training of two GANs, the BGAN loss is also minimized
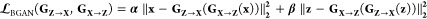
5where α and β are two constant coefficients set to 10.0 by default. The BGAN loss aims to minimize the mean-squared distance between a data point and its reconstruction by BGAN. The total training loss for the generator and discriminator networks combines adversarial training losses and BGAN loss
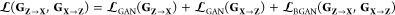
6
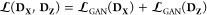
7

The iterative optimization for the BGAN model is represented by
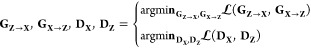
8

Upon convergence, the pseudomolecular configurations generated by the BGAN model are used to compute the entropy profiles.

## Data Availability

The code of BGAN-EPS can be found at https://github.com/rshin1209/bgan_eps/. The data set and outputs of BGAN-EPS for reproduction can be found at 10.5281/zenodo.12729645.

## Supporting Information Available

Entropy decomposition analysis (graph) and entropy profiles (table) and energy profiles (table) of diene/triene cycloaddition, tethered-diene/triene cycloaddition, NgnD-catalyzed Diels–Alder reaction, and SpnF-catalyzed Diels–Alder reaction; parameters of quasiclassical trajectory simulation; and parameters and hyperparameters of BGAN-EPS (PDF)
