# Supplementary material for: Interplay between Energy and Entropy Mediates Ambimodal Selectivity of Cycloadditions
Source: J Chem Theory Comput. 2024 Dec 6;20(24):10942–51. doi: 10.1021/acs.jctc.4c01138 (PMC11673114; doi:10.1021/acs.jctc.4c01138)
Supplement: Supplementary file 1 — ct4c01138_si_001.pdf [file ct4c01138_si_001.pdf]

Supporting Information: Interplay between Energy and Entropy Mediates Ambimodal Selectivity of Cycloadditions

Wook Shin[1], Yaning Hou[6], Xin Wang*[6], and Zhongyue J. Yang*[1-5]

[1]*Department of Chemistry, Vanderbilt University, Nashville, Tennessee 37235, United States*

[2]*Center for Structural Biology, Vanderbilt University, Nashville, Tennessee 37235, United States*

[3]*Vanderbilt Institute of Chemical Biology, Vanderbilt University, Nashville, Tennessee 37235, United States* [4]*Department of Chemical and Biomolecular Engineering, Vanderbilt University, Nashville, Tennessee 37235, United States* [5]*Data Science Institute, Vanderbilt University, Nashville, Tennessee 37235, United States* [6]*Henan-Macquarie University Joint Centre for Biomedical Innovation, School of Life Sciences, Henan University, Kaifeng, Henan, 475004, China*

Contents

Figure S1 Benchmark of BGAN-EPS Page S2
Figure S2 Comparison of Energy Profiles from the Intrinsic Reaction Coordinate (IRC) and Trajectory-Derived Data. Page S2
Figure S3 Percentage of Roaming Species vs. Change in Bond Length. Page S3
Figure S4 Time vs. Change of Bond Length for a trajectory in the diene/triene cycloaddition Page S3
Figure S5 Entropy decomposition analysis of diene/triene cycloaddition Page S4
Figure S6 Entropy decomposition analysis of tethered-diene/triene cycloaddition Page S4
Figure S7 Entropy decomposition analysis of NgnD-catalyzed Diels–Alder reaction Page S5
Figure S8 Entropy decomposition analysis of SpnF-catalyzed Diels–Alder reaction Page S5
Figure S9 Time vs. Change of Bond Length for a trajectory in the SpnF-catalyzed Diels–Alder reaction Page S6
Figure S10 VTST calculations of P1 and P8 Page S6
Table S1 Parameters of quasiclassical trajectory simulation Page S7
Table S2 Parameters of BGAN-EPS method Page S7
Table S3 Number of structural ensembles and widths for EPS method Page S7
Table S4 Distribution of reactive trajectories Page S7
Table S5-8 The number of generated pseudo-molecular configurations in structural ensembles Page S8-10
Table S9-16 The initial and unified zero entropy values of structural ensembles Page S11-19
Table S17-20 The unified zero electronic energy values of structural ensembles Page S20-21

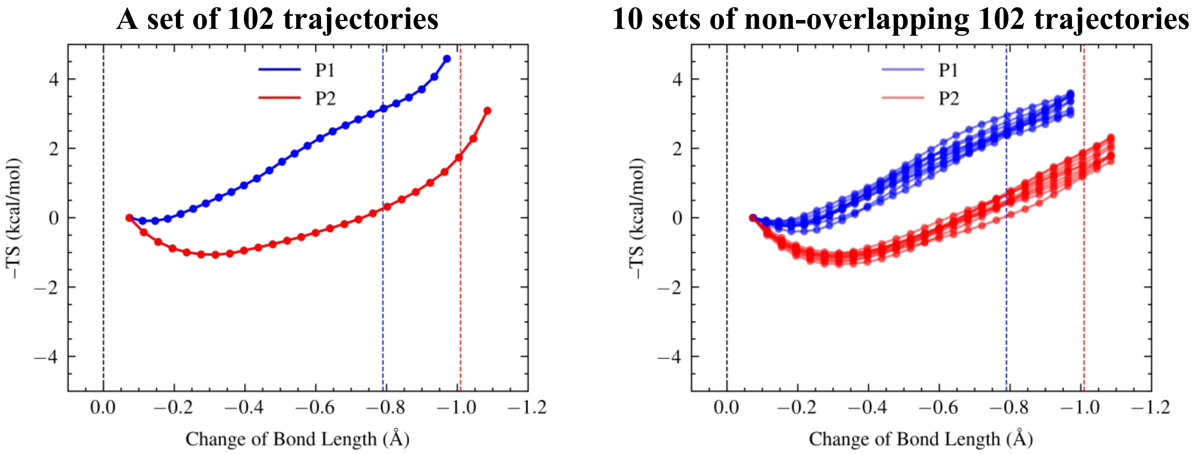

Figure S1. Comparison between the BGAN-EPS method-derived entropy profiles using a single set of 102 trajectories (left) and 10 non-overlapping sets of 102 trajectories (right) for each bifurcating product in the diene/triene cycloaddition.

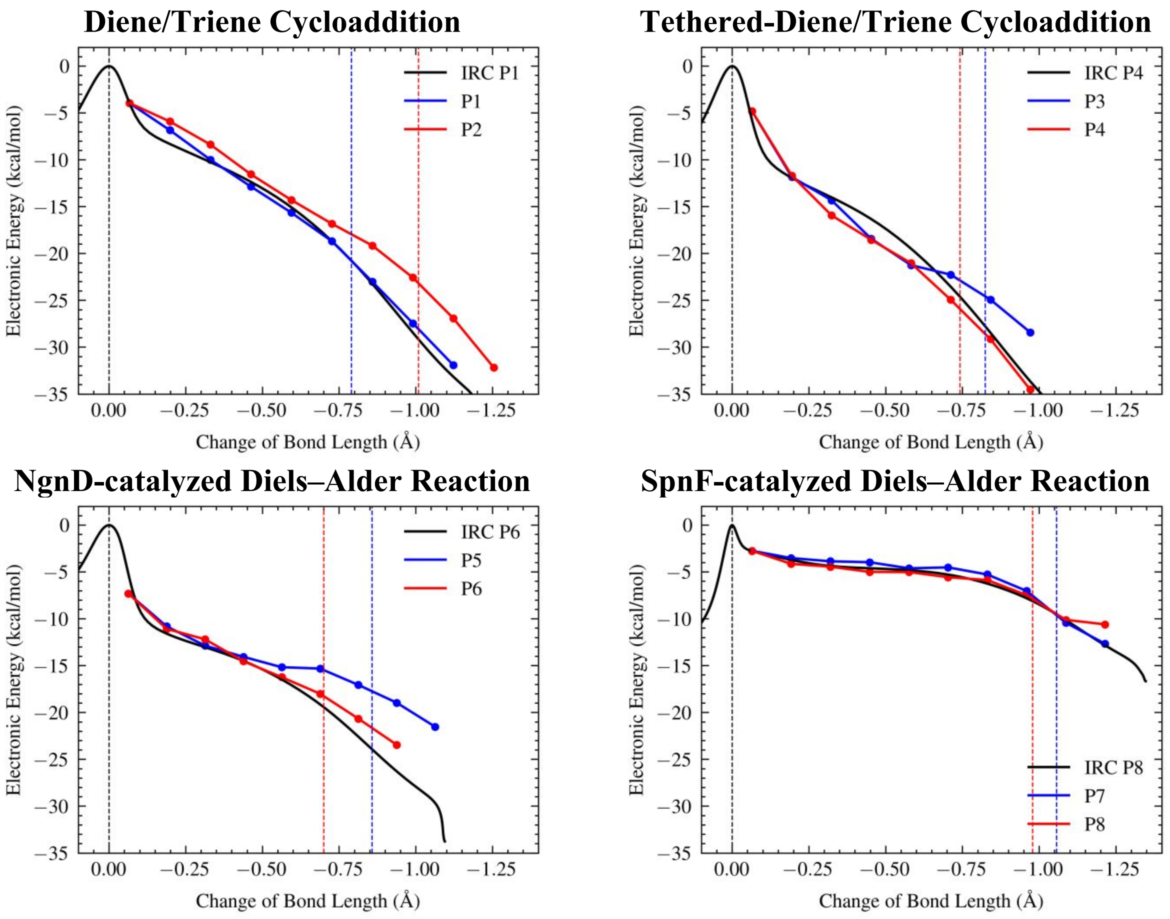

Figure S2. Comparison between the intrinsic reaction coordinate and the energy profiles derived from averaging the trajectories.

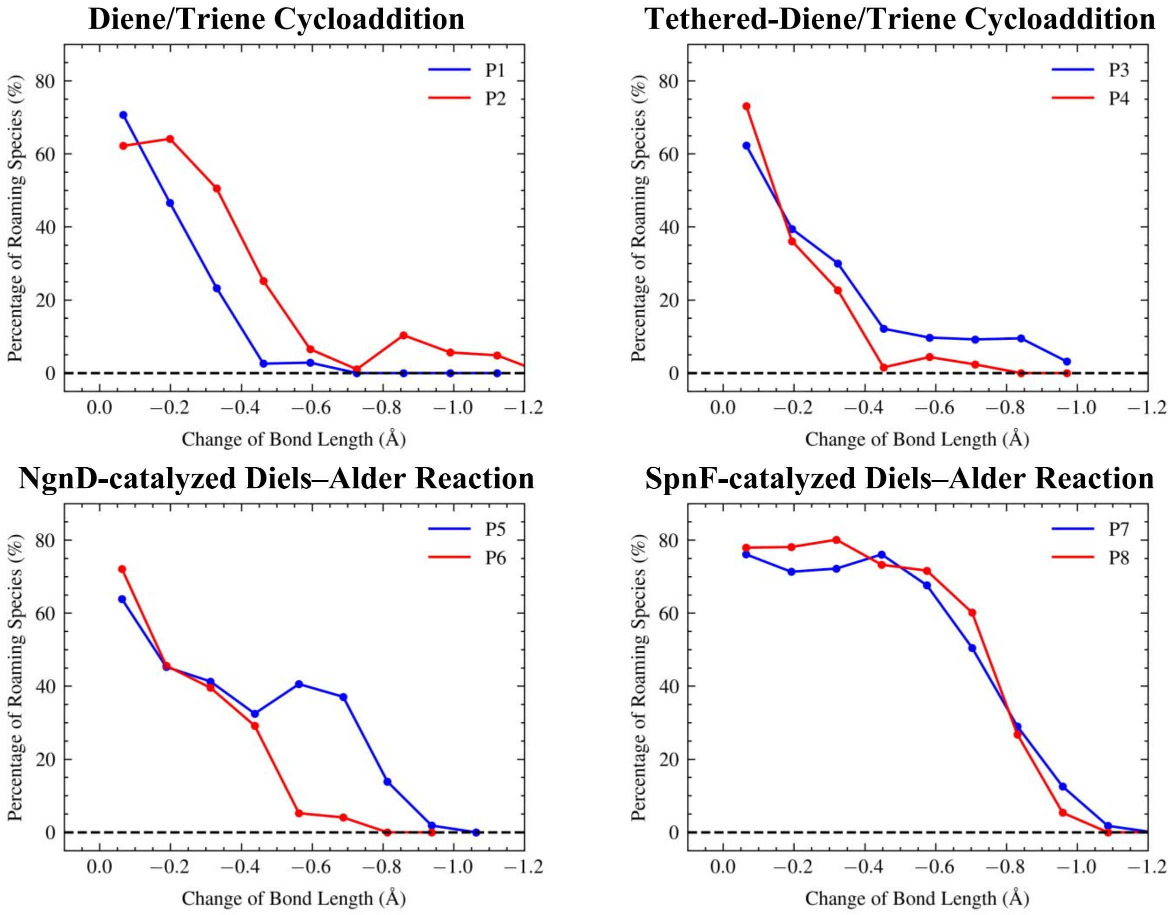

Figure S3. Percentage of roaming species vs. change in bond length. Based on the velocity of each snapshot across 102 trajectories for each bifurcating product, a range of -0.01 Å/fs < x < 0.01 Å/fs was defined as roaming, representing the population of lingering trajectories.

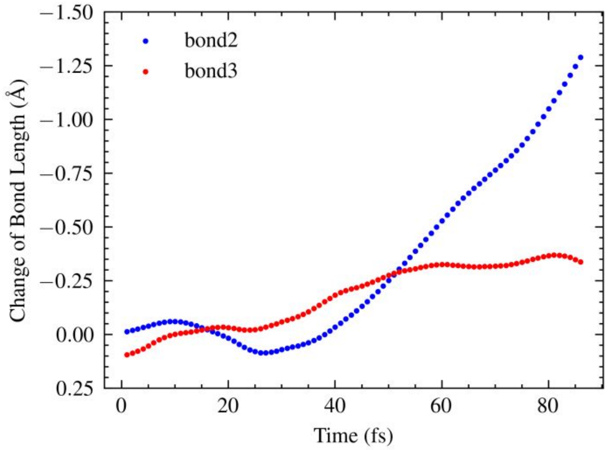

Figure S4. Time vs. change of bond length for a typical diene/triene cycloaddition trajectory that leads to the formation of P1. Bond 2 (blue) and Bond 3 (red) correspond to the potential formation for P1 and P2, respectively. Bond 2 initially roams in the vicinity of the TS region due to the influence of an entropic trap. Between 20 fs and 40 fs, it appears that the trajectory heads toward the formation of Bond 3, but after 60 fs, Bond 2 escapes from this trap and progresses to form P1.

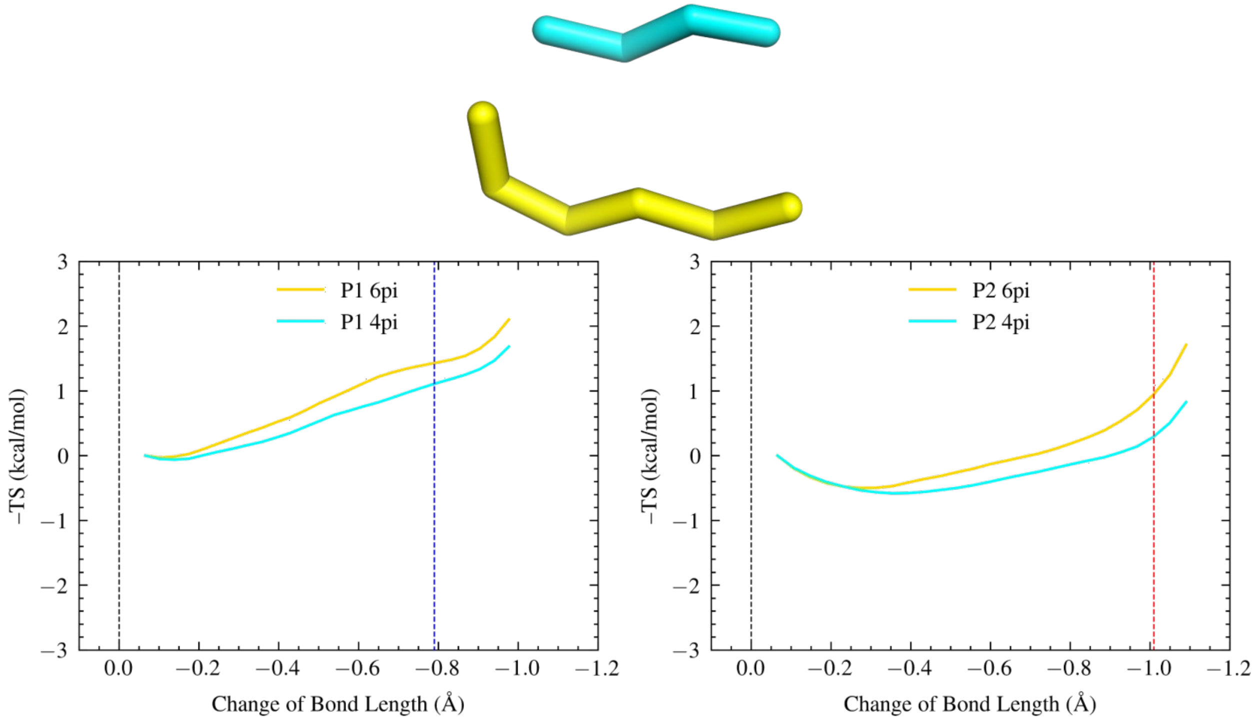

Figure S5. Entropy decomposition analysis of P1 (left) and P2 (right) in the diene/triene cycloaddition. Local structural moieties include: 4π moiety (cyan) and 6π moiety (gold).

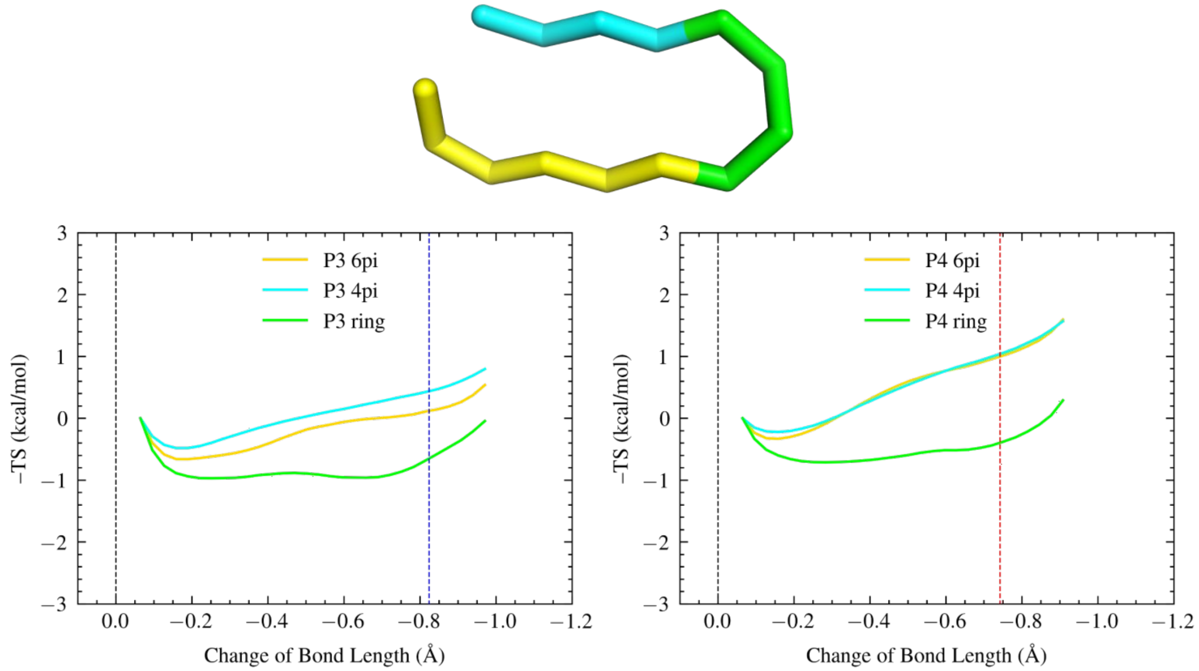

Figure S6. Entropy decomposition analysis of P3 (left) and P4 (right) in the tethered-diene/triene cycloaddition. Local structural moieties include: 4π moiety (cyan), 6π moiety (gold), and butyl tether (green).

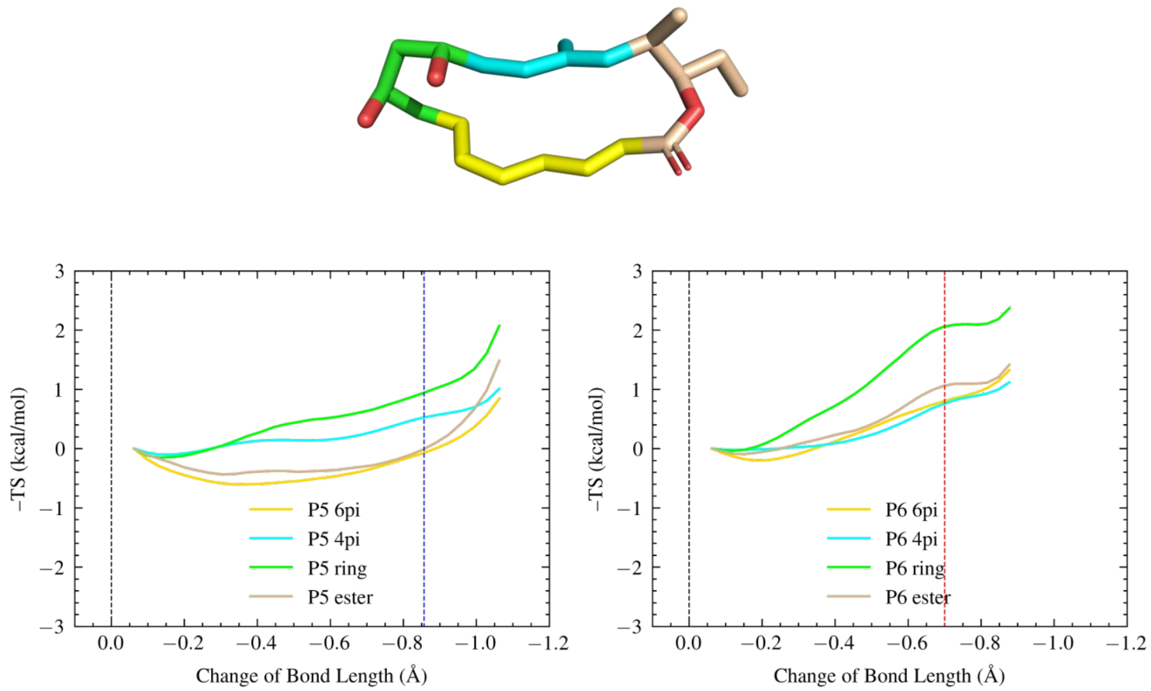

Figure S7. Entropy decomposition analysis of P5 (left) and P6 (right) in the NgnD-catalyzed Diels–Alder reaction. Local structural moieties include: 4π moiety (cyan), 6π moiety (gold), butyl tether (green), and ester tether (tan).

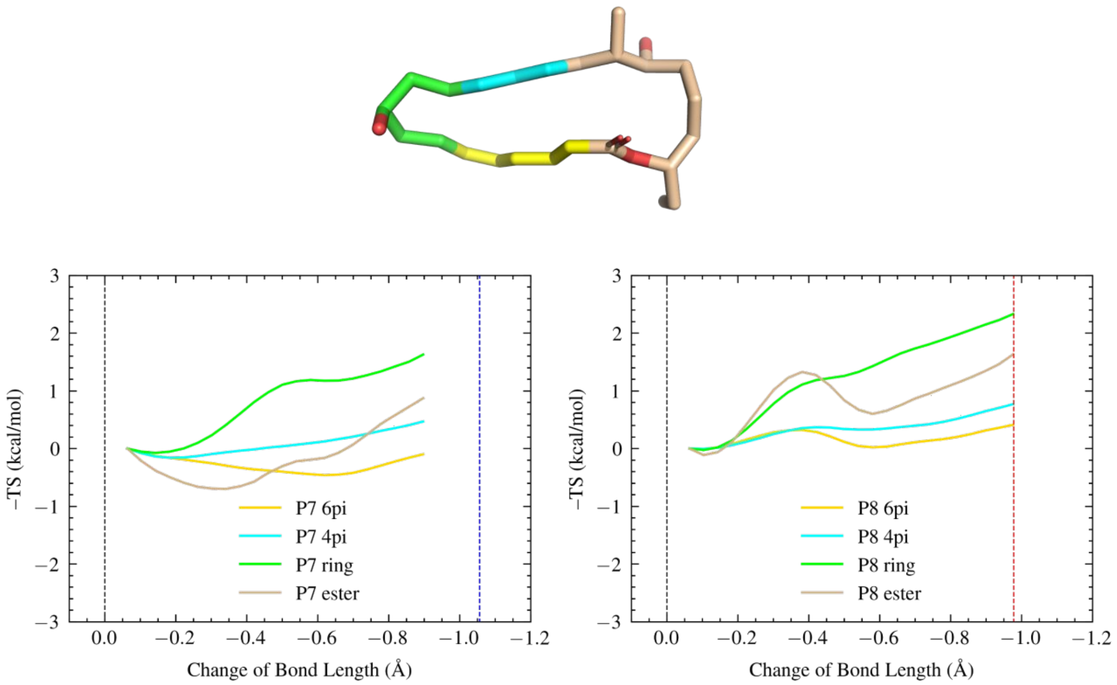

Figure S8. Entropy decomposition analysis of P7 (left) and P8 (right) in the SpnF-catalyzed Diels–Alder reaction. Local structural moieties include: 4π moiety (cyan), 6π moiety (gold), propyl tether (green), and ester tether (tan).

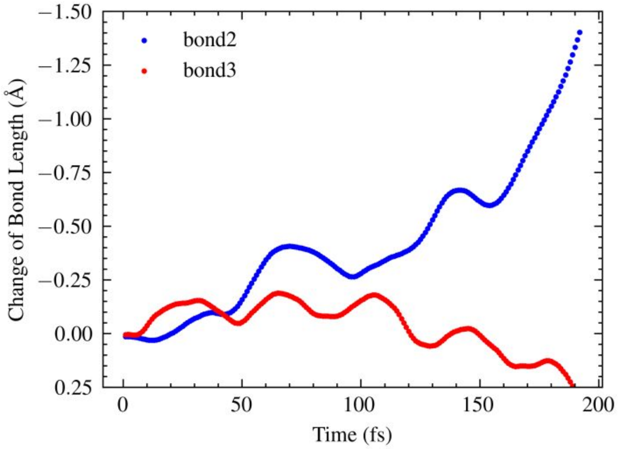

Figure S9. Time vs. change of bond length for a SpnF-catalyzed Diels–Alder reaction trajectory that leads to the formation of P7. Bond 2 (blue) and Bond 3 (red) correspond to the formation pathway for P7 and P8, respectively. Initially, Bond 3 shows an attempt to react; however, the entropic barrier inhibits this progression, causing Bond 3 to roam near the TS region. Consequently, Bond 2 proceeds to complete the reaction, although it experiences partial elongations due to the influence of an entropic trap.

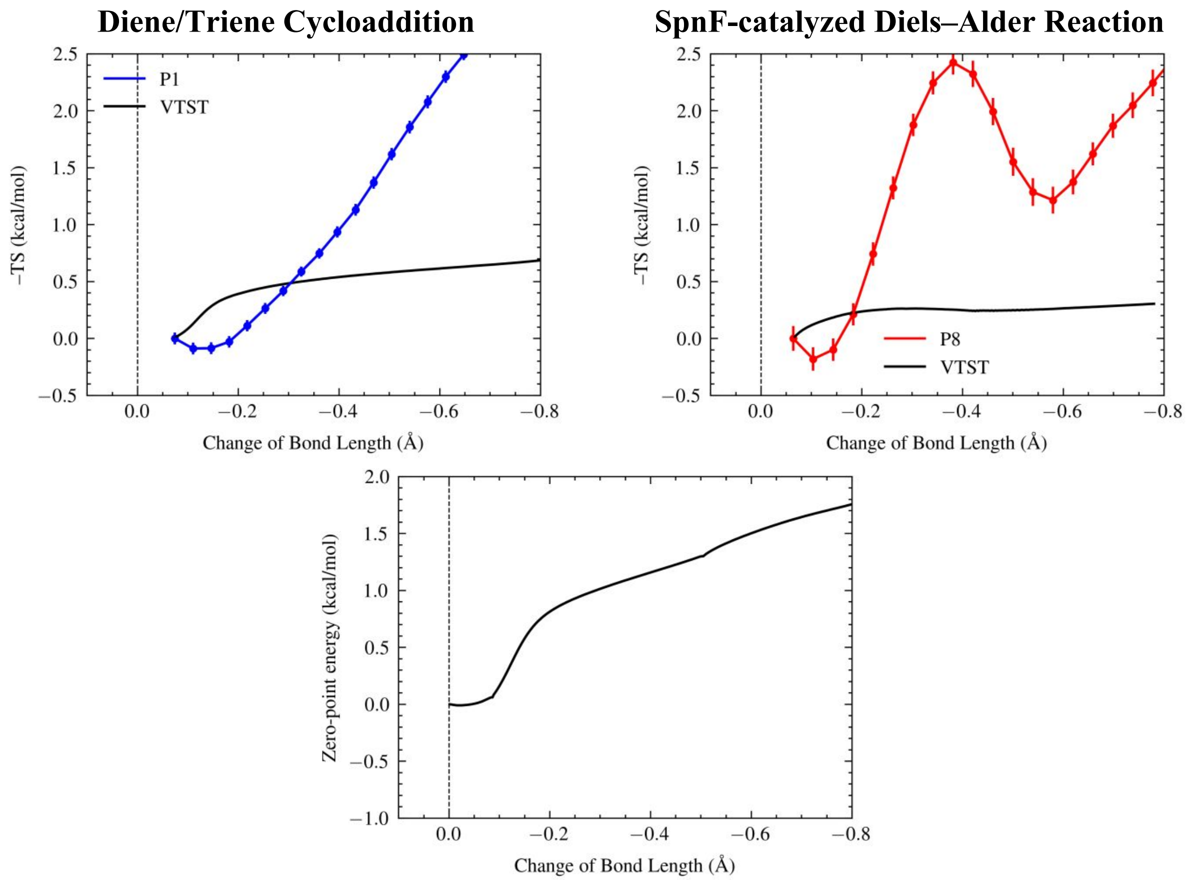

Figure S10. Comparison of P1 and P8 Entropy Profiles Derived from VTST and BGAN-EPS Methods (*top*). Zero-point energy profiles of P1 derived from VTST (*bottom*).

Table S1. The parameters used for the quasiclassical trajectory simulation.

Parameter	Value
Method/Basis Set	**B3LYP-D3/6-31G(d)**
Simulation Timestep	**1 fs**
Bond Length Formation Criterion	**1.7 Å (C-C)**
Temperature	**298.15 K**
Software Used	**Progdyn / Gaussian16**

Table S2. The parameters used for the BGAN model training.

Parameter	Value
Batch Size	**64**
Learning Rate	**1e-4**
Adam Optimizer Momentum ($\boldsymbol{\beta_1}$,$\boldsymbol{\beta_2}$)	**(0.5, 0.999)**
Epochs	**50**
Number of Entropy Evaluation Loops	**20**

Table S3. The number of structural ensembles and ensemble width for energy and entropy profiles. Moving average strategy only applies to entropy profiles.

Adduct	Ensemble Width (Å)	Number of Ensembles	Number of Moving Averages
**Diene/Triene Cycloaddition**			
[4+2]-adduct	**0.132**	**9**	**30**
[6+4]-adduct	**0.132**	**10**	**30**
**Tethered-Diene/Triene Cycloaddition**			
[4+2]-adduct	**0.129**	**8**	**30**
[6+4]-adduct	**0.129**	**8**	**30**
**NgnD-catalyzed Diels–Alder Reaction**			
[4+2]-adduct	**0.125**	**9**	**30**
[6+4]-adduct	**0.125**	**8**	**30**
**SpnF-catalyzed Diels–Alder Reacction**			
[4+2]-adduct	**0.128**	**10**	**30**
[6+4]-adduct	**0.128**	**10**	**30**

Table S4. The distribution of reactive trajectories.

Reaction	[4+2]-adduct (%)	[6+4]-adduct (%)	Recrossing (%)
Diene/triene cycloaddition	**7377 (74)**	**1961 (20)**	**583 (6)**
Tethered-diene/triene cycloaddition	**102 (33)**	**197 (63)**	**12 (4)**
NgnD-catalyzed Diels–Alder reaction	**117 (19)**	**370 (59)**	**141 (22)**
SpnF-catalyzed Diels–Alder reaction	**132 (33)**	**121 (30)**	**144 (36)**

Table S5. Specific bond range for structural ensembles and the number of generated samples per ensemble for the diene/triene cycloaddition. Percentages are calculated relative to the highest populated structural ensemble. The ensembles labeled in red font indicate the eliminated ensembles from entropy profiling because the number of samples is below 25 %.

[4+2]-adduct		[6+4]-adduct	
**Bond Range (Å)**	**Number of Samples (%)**	**Bond Range (Å)**	**Number of Samples (%)**
(2.790, 2.642]	54320 (100.00)	(3.009, 2.862]	35428 (85.88)
(2.754, 2.606]	50402 (92.79)	(2.969, 2.822]	38849 (94.18)
(2.718, 2.570]	45564 (83.88)	(2.928, 2.781]	40745 (98.77)
(2.682, 2.534]	40876 (75.25)	(2.888, 2.741]	41252 (100.00)
(2.647, 2.498]	36319 (66.86)	(2.847, 2.700]	40629 (98.49)
(2.611, 2.462]	32439 (59.72)	(2.807, 2.660]	39183 (94.98)
(2.575, 2.426]	29104 (53.58)	(2.766, 2.620]	37376 (90.60)
(2.539, 2.391]	26217 (48.27)	(2.726, 2.579]	35405 (85.83)
(2.503, 2.355]	23705 (43.64)	(2.685, 2.539]	33624 (81.51)
(2.467, 2.319]	21546 (39.67)	(2.645, 2.498]	31931 (77.41)
(2.432, 2.283]	19665 (36.20)	(2.604, 2.458]	30365 (73.61)
(2.396, 2.247]	18032 (33.20)	(2.564, 2.417]	28671 (69.50)
(2.360, 2.211]	16737 (30.81)	(2.523, 2.377]	26947 (65.32)
(2.324, 2.176]	15673 (28.85)	(2.483, 2.336]	25068 (60.77)
(2.288, 2.140]	14851 (27.34)	(2.443, 2.296]	23175 (56.18)
(2.252, 2.104]	14270 (26.27)	(2.402, 2.255]	21431 (51.95)
(2.216, 2.068]	13938 (25.66)	(2.362, 2.215]	19975 (48.42)
(2.181, 2.032]	13735 (25.29)	(2.321, 2.175]	18946 (45.93)
(2.145, 1.996]	13702 (25.22)	(2.281, 2.134]	18334 (44.44)
(2.109, 1.960]	13852 (25.50)	(2.240, 2.094]	18032 (43.71)
(2.073, 1.925]	14158 (26.06)	(2.200, 2.053]	17912 (43.42)
(2.037, 1.889]	14567 (26.82)	(2.159, 2.013]	17709 (42.93)
(2.001, 1.853]	15005 (27.62)	(2.119, 1.972]	17345 (42.05)
(1.966, 1.817]	15271 (28.11)	(2.078, 1.932]	16526 (40.06)
(1.930, 1.781]	15041 (27.69)	(2.038, 1.891]	15032 (36.44)
(1.894, 1.745]	13872 (25.54)	(1.998, 1.851]	12537 (30.39)
(1.858, 1.710]	11279 (20.76)	(1.957, 1.810]	8938 (21.67)
(1.822, 1.674]	7662 (14.11)	(1.917, 1.770]	5112 (12.39)
(1.786, 1.638]	4095 (7.54)	(1.876, 1.729]	2161 (5.24)
(1.751, 1.602]	1605 (2.96)	(1.836, 1.689]	672 (1.63)

Table S6. Specific bond range for structural ensembles and the number of generated samples per ensemble for the tethered-diene/triene cycloaddition. Percentages are calculated relative to the highest populated structural ensemble. The ensembles labeled in red font indicate the eliminated ensembles from entropy profiling because the number of samples is below 25 %.

[4+2]-adduct		[6+4]-adduct	
**Bond Range (Å)**	**Number of Samples (%)**	**Bond Range (Å)**	**Number of Samples (%)**
(2.824, 2.695]	46618 (100.00)	(2.742, 2.613]	51783 (100.00)
(2.793, 2.663]	45295 (97.16)	(2.711, 2.581]	49005 (94.63)
(2.762, 2.632]	43176 (92.62)	(2.680, 2.550]	45664 (88.18)
(2.730, 2.601]	40564 (87.01)	(2.648, 2.519]	42015 (81.14)
(2.699, 2.570]	37845 (81.18)	(2.617, 2.488]	38455 (74.26)
(2.668, 2.538]	35326 (75.78)	(2.586, 2.456]	35195 (67.97)
(2.637, 2.507]	32878 (70.53)	(2.555, 2.425]	32229 (62.24)
(2.605, 2.476]	30722 (65.90)	(2.523, 2.394]	29564 (57.09)
(2.574, 2.445]	28820 (61.82)	(2.492, 2.363]	27203 (52.53)
(2.543, 2.414]	27089 (58.11)	(2.461, 2.332]	25076 (48.43)
(2.512, 2.382]	25650 (55.02)	(2.430, 2.300]	23254 (44.91)
(2.480, 2.351]	24379 (52.30)	(2.398, 2.269]	21668 (41.84)
(2.449, 2.320]	23273 (49.92)	(2.367, 2.238]	20270 (39.14)
(2.418, 2.289]	22407 (48.07)	(2.336, 2.207]	19172 (37.02)
(2.387, 2.257]	21618 (46.37)	(2.305, 2.175]	18244 (35.23)
(2.356, 2.226]	20975 (44.99)	(2.274, 2.144]	17515 (33.82)
(2.324, 2.195]	20490 (43.95)	(2.242, 2.113]	16953 (32.74)
(2.293, 2.164]	20125 (43.17)	(2.211, 2.082]	16495 (31.86)
(2.262, 2.133]	19876 (42.64)	(2.180, 2.051]	16130 (31.15)
(2.231, 2.101]	19835 (42.55)	(2.149, 2.019]	15767 (30.45)
(2.199, 2.070]	19845 (42.57)	(2.117, 1.988]	15442 (29.82)
(2.168, 2.039]	19900 (42.69)	(2.086, 1.957]	15160 (29.28)
(2.137, 2.008]	19963 (42.82)	(2.055, 1.926]	14952 (28.87)
(2.106, 1.976]	19962 (42.82)	(2.024, 1.894]	14855 (28.69)
(2.075, 1.945]	19866 (42.61)	(1.993, 1.863]	14810 (28.60)
(2.043, 1.914]	19715 (42.29)	(1.961, 1.832]	14678 (28.34)
(2.012, 1.883]	19532 (41.90)	(1.930, 1.801]	14337 (27.69)
(1.981, 1.851]	19240 (41.27)	(1.899, 1.769]	13471 (26.01)
(1.950, 1.820]	18759 (40.24)	(1.868, 1.738]	11745 (22.68)
(1.918, 1.789]	17854 (38.30)	(1.836, 1.707]	9142 (17.66)

Table S7. Specific bond range for structural ensembles and the number of generated samples per ensemble for the NgnD-catalyzed Diels–Alder reaction. Percentages are calculated relative to the highest populated structural ensemble. The ensembles labeled in red font indicate the eliminated ensembles from entropy profiling because the number of samples is below 25 %.

[4+2]-adduct		[6+4]-adduct	
**Bond Range (Å)**	**Number of Samples (%)**	**Bond Range (Å)**	**Number of Samples (%)**
(2.857, 2.732]	50772 (100.00)	(2.700, 2.575]	59892 (100.00)
(2.823, 2.698]	48641 (95.80)	(2.670, 2.545]	56189 (93.82)
(2.788, 2.663]	45521 (89.66)	(2.640, 2.515]	51918 (86.69)
(2.754, 2.629]	42238 (83.19)	(2.609, 2.484]	47747 (79.72)
(2.719, 2.594]	39248 (77.30)	(2.579, 2.454]	43900 (73.30)
(2.685, 2.560]	36560 (72.01)	(2.549, 2.424]	40618 (67.82)
(2.650, 2.525]	34332 (67.62)	(2.519, 2.394]	37817 (63.14)
(2.616, 2.491]	32705 (64.42)	(2.489, 2.364]	35366 (59.05)
(2.581, 2.456]	31519 (62.08)	(2.459, 2.334]	33153 (55.36)
(2.547, 2.422]	30785 (60.63)	(2.428, 2.303]	31164 (52.03)
(2.512, 2.387]	30442 (59.96)	(2.398, 2.273]	29344 (49.00)
(2.478, 2.353]	30353 (59.78)	(2.368, 2.243]	27529 (45.97)
(2.443, 2.318]	30322 (59.72)	(2.338, 2.213]	25844 (43.15)
(2.409, 2.284]	30370 (59.82)	(2.308, 2.183]	24226 (40.45)
(2.374, 2.249]	30217 (59.52)	(2.278, 2.153]	22701 (37.90)
(2.340, 2.215]	30009 (59.11)	(2.247, 2.122]	21245 (35.47)
(2.305, 2.180]	29710 (58.52)	(2.217, 2.092]	19936 (33.29)
(2.271, 2.146]	29215 (57.54)	(2.187, 2.062]	18740 (31.29)
(2.236, 2.111]	28670 (56.47)	(2.157, 2.032]	17797 (29.72)
(2.202, 2.077]	27993 (55.14)	(2.127, 2.002]	17131 (28.60)
(2.167, 2.042]	27128 (53.43)	(2.097, 1.972]	16721 (27.92)
(2.133, 2.008]	26157 (51.52)	(2.066, 1.941]	16590 (27.70)
(2.098, 1.973]	25128 (49.49)	(2.036, 1.911]	16647 (27.80)
(2.064, 1.939]	24108 (47.48)	(2.006, 1.881]	16796 (28.04)
(2.029, 1.904]	23278 (45.85)	(1.976, 1.851]	16989 (28.37)
(1.995, 1.870]	22696 (44.70)	(1.946, 1.821]	17016 (28.41)
(1.960, 1.835]	22227 (43.78)	(1.916, 1.791]	16693 (27.87)
(1.926, 1.801]	21389 (42.13)	(1.885, 1.760]	15612 (26.07)
(1.891, 1.766]	19412 (38.23)	(1.855, 1.730]	13470 (22.49)
(1.857, 1.732]	15882 (31.28)	(1.825, 1.700]	10332 (17.25)

Table S8. Specific bond range for structural ensembles and the number of generated samples per ensemble for the SpnF-catalyzed Diels–Alder reaction. Percentages are calculated relative to the highest populated structural ensemble. The ensembles labeled in red font indicate the eliminated ensembles from entropy profiling because the number of samples is below 25 %.

**[4+2]-adduct**		**[6+4]-adduct**	
**Bond Range (Å)**	**Number of Samples (%)**	**Bond Range (Å)**	**Number of Samples (%)**
(3.056, 2.928]	106056 (95.79)	(2.978, 2.850]	110506 (100.00)
(3.016, 2.889]	110080 (99.43)	(2.938, 2.811]	109692 (99.26)
(2.977, 2.849]	110714 (100.00)	(2.899, 2.771]	107690 (97.45)
(2.937, 2.809]	108709 (98.19)	(2.859, 2.731]	105087 (95.10)
(2.897, 2.770]	105411 (95.21)	(2.819, 2.692]	101667 (92.00)
(2.858, 2.730]	101222 (91.43)	(2.780, 2.652]	97051 (87.82)
(2.818, 2.690]	96733 (87.37)	(2.740, 2.612]	91188 (82.52)
(2.778, 2.651]	92263 (83.33)	(2.700, 2.573]	84576 (76.54)
(2.739, 2.611]	87706 (79.22)	(2.661, 2.533]	77747 (70.36)
(2.699, 2.571]	82938 (74.91)	(2.621, 2.493]	71157 (64.39)
(2.659, 2.532]	77611 (70.10)	(2.581, 2.454]	65130 (58.94)
(2.620, 2.492]	71805 (64.86)	(2.542, 2.414]	60099 (54.39)
(2.580, 2.452]	65535 (59.19)	(2.502, 2.374]	56337 (50.98)
(2.540, 2.413]	59165 (53.44)	(2.462, 2.335]	53509 (48.42)
(2.501, 2.373]	53344 (48.18)	(2.423, 2.295]	51254 (46.38)
(2.461, 2.333]	48073 (43.42)	(2.383, 2.255]	48966 (44.31)
(2.421, 2.294]	43656 (39.43)	(2.343, 2.216]	46346 (41.94)
(2.382, 2.254]	39822 (35.97)	(2.304, 2.176]	43361 (39.24)
(2.342, 2.214]	36587 (33.05)	(2.264, 2.136]	40036 (36.23)
(2.302, 2.175]	33734 (30.47)	(2.224, 2.097]	36715 (33.22)
(2.263, 2.135]	31254 (28.23)	(2.185, 2.057]	33686 (30.48)
(2.223, 2.095]	28975 (26.17)	(2.145, 2.017]	31179 (28.21)
(2.183, 2.056]	27086 (24.47)	(2.105, 1.978]	29302 (26.52)
(2.144, 2.016]	25510 (23.04)	(2.066, 1.938]	27911 (25.26)
(2.104, 1.976]	24277 (21.93)	(2.026, 1.898]	26601 (24.07)
(2.064, 1.937]	23347 (21.09)	(1.986, 1.859]	24539 (22.21)
(2.025, 1.897]	22532 (20.35)	(1.947, 1.819]	20939 (18.95)
(1.985, 1.857]	21259 (19.20)	(1.907, 1.779]	15669 (14.18)
(1.945, 1.818]	18646 (16.84)	(1.867, 1.740]	9617 (8.70)
(1.906, 1.778]	14066 (12.71)	(1.828, 1.700]	4588 (4.15)

Table S9. Specific bond range for structural ensembles and the initial total configurational entropy calculated in each structural ensemble for the **diene/triene cycloaddition**. The mean and standard error were calculated from 20 rounds of BGAN-EPS. The ensembles labeled in red font indicate the eliminated ensembles from entropy profiling because the number of samples is below 25 %.

[4+2]-adduct		[6+4]-adduct	
**Bond Range (Å)**	$-TS\ (kcal\ /\ mol)$	**Bond Range (Å)**	$-TS\ (kcal\ /\ mol)$
(2.790, 2.642]	30.78 ± 0.05	(3.009, 2.862]	31.54 ± 0.06
(2.754, 2.606]	30.69 ± 0.05	(2.969, 2.822]	31.12 ± 0.06
(2.718, 2.570]	30.69 ± 0.05	(2.928, 2.781]	30.85 ± 0.05
(2.682, 2.534]	30.75 ± 0.05	(2.888, 2.741]	30.66 ± 0.05
(2.647, 2.498]	30.89 ± 0.05	(2.847, 2.700]	30.54 ± 0.05
(2.611, 2.462]	31.04 ± 0.05	(2.807, 2.660]	30.48 ± 0.05
(2.575, 2.426]	31.20 ± 0.05	(2.766, 2.620]	30.47 ± 0.06
(2.539, 2.391]	31.37 ± 0.04	(2.726, 2.579]	30.50 ± 0.06
(2.503, 2.355]	31.53 ± 0.05	(2.685, 2.539]	30.59 ± 0.06
(2.467, 2.319]	31.72 ± 0.05	(2.645, 2.498]	30.68 ± 0.06
(2.432, 2.283]	31.91 ± 0.05	(2.604, 2.458]	30.77 ± 0.06
(2.396, 2.247]	32.15 ± 0.05	(2.564, 2.417]	30.88 ± 0.06
(2.360, 2.211]	32.40 ± 0.06	(2.523, 2.377]	30.99 ± 0.06
(2.324, 2.176]	32.64 ± 0.06	(2.483, 2.336]	31.11 ± 0.06
(2.288, 2.140]	32.86 ± 0.06	(2.443, 2.296]	31.23 ± 0.06
(2.252, 2.104]	33.08 ± 0.05	(2.402, 2.255]	31.36 ± 0.06
(2.216, 2.068]	33.27 ± 0.05	(2.362, 2.215]	31.49 ± 0.05
(2.181, 2.032]	33.45 ± 0.05	(2.321, 2.175]	31.67 ± 0.05
(2.145, 1.996]	33.62 ± 0.05	(2.281, 2.134]	31.86 ± 0.05
(2.109, 1.960]	33.77 ± 0.06	(2.240, 2.094]	32.07 ± 0.05
(2.073, 1.925]	33.93 ± 0.07	(2.200, 2.053]	32.28 ± 0.05
(2.037, 1.889]	34.08 ± 0.07	(2.159, 2.013]	32.55 ± 0.05
(2.001, 1.853]	34.25 ± 0.07	(2.119, 1.972]	32.86 ± 0.06
(1.966, 1.817]	34.49 ± 0.07	(2.078, 1.932]	33.28 ± 0.06
(1.930, 1.781]	34.85 ± 0.07	(2.038, 1.891]	33.82 ± 0.07
(1.894, 1.745]	35.37 ± 0.07	(1.998, 1.851]	34.63 ± 0.07
(1.858, 1.710]	36.20 ± 0.08	(1.957, 1.810]	35.93 ± 0.08
(1.822, 1.674]	37.55 ± 0.11	(1.917, 1.770]	38.40 ± 0.15
(1.786, 1.638]	40.32 ± 0.24	(1.876, 1.729]	44.41 ± 0.39
(1.751, 1.602]	48.18 ± 0.80	(1.836, 1.689]	60.73 ± 0.81

Table S10. Specific range of change of bond lengths for structural ensembles relative to the respective bond lengths in the transition state structure and the unified zero (take the first point as zero point) total configurational entropy calculated in each structural ensemble for the diene/triene cycloaddition. The mean and standard error were calculated from 20 rounds of BGAN-EPS. The ensembles labeled in red font indicate the eliminated ensembles from entropy profiling because the number of samples is below 25 %.

[4+2]-adduct		[6+4]-adduct	
**Bond Range (Å)**	$-TS\ (kcal\ /\ mol)$	**Bond Range (Å)**	$-TS\ (kcal\ /\ mol)$
(0.000, -0.148]	0.00 ± 0.05	(0.000, -0.147]	0.00 ± 0.06
(-0.036, -0.184]	-0.09 ± 0.05	(-0.040, -0.187]	-0.42 ± 0.06
(-0.072, -0.220]	-0.09 ± 0.05	(-0.081, -0.228]	-0.69 ± 0.05
(-0.108, -0.256]	-0.03 ± 0.05	(-0.121, -0.268]	-0.88 ± 0.05
(-0.143, -0.292]	0.11 ± 0.05	(-0.162, -0.309]	-0.99 ± 0.05
(-0.179, -0.328]	0.26 ± 0.05	(-0.202, -0.349]	-1.06 ± 0.05
(-0.215, -0.364]	0.42 ± 0.05	(-0.243, -0.389]	-1.06 ± 0.06
(-0.251, -0.399]	0.59 ± 0.04	(-0.283, -0.430]	-1.03 ± 0.06
(-0.287, -0.435]	0.75 ± 0.05	(-0.324, -0.470]	-0.95 ± 0.06
(-0.323, -0.471]	0.94 ± 0.05	(-0.364, -0.511]	-0.86 ± 0.06
(-0.358, -0.507]	1.13 ± 0.05	(-0.405, -0.551]	-0.76 ± 0.06
(-0.394, -0.543]	1.37 ± 0.05	(-0.445, -0.592]	-0.66 ± 0.06
(-0.430, -0.579]	1.62 ± 0.06	(-0.486, -0.632]	-0.55 ± 0.06
(-0.466, -0.614]	1.86 ± 0.06	(-0.526, -0.673]	-0.43 ± 0.06
(-0.502, -0.650]	2.08 ± 0.06	(-0.566, -0.713]	-0.31 ± 0.06
(-0.538, -0.686]	2.30 ± 0.05	(-0.607, -0.754]	-0.18 ± 0.06
(-0.574, -0.722]	2.49 ± 0.05	(-0.647, -0.794]	-0.04 ± 0.05
(-0.609, -0.758]	2.67 ± 0.05	(-0.688, -0.834]	0.13 ± 0.05
(-0.645, -0.794]	2.84 ± 0.05	(-0.728, -0.875]	0.32 ± 0.05
(-0.681, -0.830]	2.99 ± 0.06	(-0.769, -0.915]	0.53 ± 0.05
(-0.717, -0.865]	3.15 ± 0.07	(-0.809, -0.956]	0.75 ± 0.05
(-0.753, -0.901]	3.30 ± 0.07	(-0.850, -0.996]	1.02 ± 0.05
(-0.789, -0.937]	3.47 ± 0.07	(-0.890, -1.037]	1.32 ± 0.06
(-0.824, -0.973]	3.71 ± 0.07	(-0.931, -1.077]	1.74 ± 0.06
(-0.860, -1.009]	4.07 ± 0.07	(-0.971, -1.118]	2.28 ± 0.07
(-0.896, -1.045]	4.59 ± 0.07	(-1.011, -1.158]	3.09 ± 0.07
(-0.932, -1.080]	5.42 ± 0.08	(-1.052, -1.199]	4.39 ± 0.08
(-0.968, -1.116]	6.78 ± 0.11	(-1.092, -1.239]	6.86 ± 0.15
(-1.004, -1.152]	9.54 ± 0.24	(-1.133, -1.280]	12.87 ± 0.39
(-1.039, -1.188]	17.40 ± 0.80	(-1.173, -1.320]	29.20 ± 0.81

Table S11. Specific bond range for structural ensembles and the initial total configurational entropy calculated in each structural ensemble for the tethered-diene/triene cycloaddition. The mean and standard error were calculated from 20 rounds of BGAN-EPS. The ensembles labeled in red font indicate the eliminated ensembles from entropy profiling because the number of samples is below 25 %.

[4+2]-adduct		[6+4]-adduct	
**Bond Range (Å)**	$-TS\ (kcal\ /\ mol)$	**Bond Range (Å)**	$-TS\ (kcal\ /\ mol)$
(2.824, 2.695]	47.35 ± 0.12	(2.742, 2.613]	45.79 ± 0.12
(2.793, 2.663]	46.11 ± 0.10	(2.711, 2.581]	45.02 ± 0.09
(2.762, 2.632]	45.47 ± 0.07	(2.680, 2.550]	44.63 ± 0.07
(2.730, 2.601]	45.17 ± 0.07	(2.648, 2.519]	44.45 ± 0.06
(2.699, 2.570]	45.06 ± 0.07	(2.617, 2.488]	44.38 ± 0.06
(2.668, 2.538]	45.04 ± 0.07	(2.586, 2.456]	44.38 ± 0.05
(2.637, 2.507]	45.09 ± 0.07	(2.555, 2.425]	44.44 ± 0.05
(2.605, 2.476]	45.17 ± 0.07	(2.523, 2.394]	44.54 ± 0.05
(2.574, 2.445]	45.26 ± 0.07	(2.492, 2.363]	44.69 ± 0.04
(2.543, 2.414]	45.38 ± 0.07	(2.461, 2.332]	44.87 ± 0.05
(2.512, 2.382]	45.52 ± 0.07	(2.430, 2.300]	45.06 ± 0.05
(2.480, 2.351]	45.64 ± 0.07	(2.398, 2.269]	45.24 ± 0.06
(2.449, 2.320]	45.76 ± 0.07	(2.367, 2.238]	45.42 ± 0.06
(2.418, 2.289]	45.86 ± 0.07	(2.336, 2.207]	45.59 ± 0.07
(2.387, 2.257]	45.95 ± 0.07	(2.305, 2.175]	45.75 ± 0.07
(2.356, 2.226]	46.01 ± 0.06	(2.274, 2.144]	45.90 ± 0.07
(2.324, 2.195]	46.05 ± 0.06	(2.242, 2.113]	46.05 ± 0.06
(2.293, 2.164]	46.11 ± 0.06	(2.211, 2.082]	46.18 ± 0.06
(2.262, 2.133]	46.16 ± 0.06	(2.180, 2.051]	46.29 ± 0.06
(2.231, 2.101]	46.22 ± 0.06	(2.149, 2.019]	46.40 ± 0.06
(2.199, 2.070]	46.27 ± 0.06	(2.117, 1.988]	46.52 ± 0.06
(2.168, 2.039]	46.36 ± 0.06	(2.086, 1.957]	46.67 ± 0.06
(2.137, 2.008]	46.47 ± 0.06	(2.055, 1.926]	46.85 ± 0.06
(2.106, 1.976]	46.60 ± 0.06	(2.024, 1.894]	47.04 ± 0.06
(2.075, 1.945]	46.77 ± 0.06	(1.993, 1.863]	47.30 ± 0.06
(2.043, 1.914]	46.96 ± 0.07	(1.961, 1.832]	47.60 ± 0.06
(2.012, 1.883]	47.19 ± 0.07	(1.930, 1.801]	48.01 ± 0.06
(1.981, 1.851]	47.46 ± 0.07	(1.899, 1.769]	48.59 ± 0.07
(1.950, 1.820]	47.83 ± 0.07	(1.868, 1.738]	49.44 ± 0.08
(1.918, 1.789]	48.30 ± 0.07	(1.836, 1.707]	50.76 ± 0.10

Table S12. Specific range of change of bond lengths for structural ensembles relative to the respective bond lengths in the transition state structure and the unified zero (take the first point as zero point) total configurational entropy calculated in each structural ensemble for the tethered-diene/triene cycloaddition. The mean and standard error were calculated from 20 rounds of BGAN-EPS. The ensembles labeled in red font indicate the eliminated ensembles from entropy profiling because the number of samples is below 25 %.

[4+2]-adduct		[6+4]-adduct	
**Bond Range (Å)**	$-TS\ (kcal/mol)$	**Bond Range (Å)**	$-TS\ (kcal/mol)$
(0.000, -0.129]	0.00 ± 0.12	(0.000, -0.129]	0.00 ± 0.12
(-0.031, -0.161]	-1.23 ± 0.10	(-0.031, -0.161]	-0.77 ± 0.09
(-0.062, -0.192]	-1.87 ± 0.07	(-0.062, -0.192]	-1.16 ± 0.07
(-0.094, -0.223]	-2.17 ± 0.07	(-0.094, -0.223]	-1.34 ± 0.06
(-0.125, -0.254]	-2.28 ± 0.07	(-0.125, -0.254]	-1.41 ± 0.06
(-0.156, -0.286]	-2.30 ± 0.07	(-0.156, -0.286]	-1.41 ± 0.05
(-0.187, -0.317]	-2.25 ± 0.07	(-0.187, -0.317]	-1.35 ± 0.05
(-0.219, -0.348]	-2.17 ± 0.07	(-0.219, -0.348]	-1.24 ± 0.05
(-0.250, -0.379]	-2.08 ± 0.07	(-0.250, -0.379]	-1.09 ± 0.04
(-0.281, -0.410]	-1.96 ± 0.07	(-0.281, -0.410]	-0.92 ± 0.05
(-0.312, -0.442]	-1.83 ± 0.07	(-0.312, -0.442]	-0.73 ± 0.05
(-0.344, -0.473]	-1.71 ± 0.07	(-0.344, -0.473]	-0.55 ± 0.06
(-0.375, -0.504]	-1.58 ± 0.07	(-0.375, -0.504]	-0.37 ± 0.06
(-0.406, -0.535]	-1.48 ± 0.07	(-0.406, -0.535]	-0.20 ± 0.07
(-0.437, -0.567]	-1.40 ± 0.07	(-0.437, -0.567]	-0.04 ± 0.07
(-0.468, -0.598]	-1.34 ± 0.06	(-0.468, -0.598]	0.12 ± 0.07
(-0.500, -0.629]	-1.29 ± 0.06	(-0.500, -0.629]	0.26 ± 0.06
(-0.531, -0.660]	-1.24 ± 0.06	(-0.531, -0.660]	0.40 ± 0.06
(-0.562, -0.691]	-1.18 ± 0.06	(-0.562, -0.691]	0.50 ± 0.06
(-0.593, -0.723]	-1.13 ± 0.06	(-0.593, -0.723]	0.61 ± 0.06
(-0.625, -0.754]	-1.07 ± 0.06	(-0.625, -0.754]	0.74 ± 0.06
(-0.656, -0.785]	-0.99 ± 0.06	(-0.656, -0.785]	0.88 ± 0.06
(-0.687, -0.816]	-0.88 ± 0.06	(-0.687, -0.816]	1.06 ± 0.06
(-0.718, -0.848]	-0.75 ± 0.06	(-0.718, -0.848]	1.25 ± 0.06
(-0.749, -0.879]	-0.57 ± 0.06	(-0.749, -0.879]	1.51 ± 0.06
(-0.781, -0.910]	-0.39 ± 0.07	(-0.781, -0.910]	1.81 ± 0.06
(-0.812, -0.941]	-0.16 ± 0.07	(-0.812, -0.941]	2.22 ± 0.06
(-0.843, -0.973]	0.12 ± 0.07	(-0.843, -0.973]	2.80 ± 0.07
(-0.874, -1.004]	0.49 ± 0.07	(-0.874, -1.004]	3.65 ± 0.08
(-0.906, -1.035]	0.95 ± 0.07	(-0.906, -1.035]	4.97 ± 0.10

Table S13. Specific bond range for structural ensembles and the initial total configurational entropy calculated in each structural ensemble for the NgnD-catalyzed Diels–Alder reaction. The mean and standard error were calculated from 20 rounds of BGAN-EPS. The ensembles labeled in red font indicate the eliminated ensembles from entropy profiling because the number of samples is below 25 %.

[4+2]-adduct		[6+4]-adduct	
**Bond Range (Å)**	$-TS\ (kcal\ /\ mol)$	**Bond Range (Å)**	$-TS\ (kcal\ /\ mol)$
(2.857, 2.732]	76.29 ± 0.10	(2.700, 2.575]	75.48 ± 0.07
(2.823, 2.698]	75.79 ± 0.09	(2.670, 2.545]	75.23 ± 0.07
(2.788, 2.663]	75.46 ± 0.09	(2.640, 2.515]	75.04 ± 0.07
(2.754, 2.629]	75.26 ± 0.09	(2.609, 2.484]	74.90 ± 0.07
(2.719, 2.594]	75.10 ± 0.09	(2.579, 2.454]	74.85 ± 0.07
(2.685, 2.560]	75.02 ± 0.09	(2.549, 2.424]	74.85 ± 0.07
(2.650, 2.525]	74.99 ± 0.09	(2.519, 2.394]	74.89 ± 0.07
(2.616, 2.491]	75.01 ± 0.09	(2.489, 2.364]	74.99 ± 0.07
(2.581, 2.456]	75.08 ± 0.09	(2.459, 2.334]	75.09 ± 0.07
(2.547, 2.422]	75.20 ± 0.09	(2.428, 2.303]	75.20 ± 0.07
(2.512, 2.387]	75.29 ± 0.09	(2.398, 2.273]	75.33 ± 0.07
(2.478, 2.353]	75.39 ± 0.09	(2.368, 2.243]	75.48 ± 0.07
(2.443, 2.318]	75.46 ± 0.09	(2.338, 2.213]	75.66 ± 0.07
(2.409, 2.284]	75.48 ± 0.09	(2.308, 2.183]	75.86 ± 0.07
(2.374, 2.249]	75.53 ± 0.09	(2.278, 2.153]	76.10 ± 0.07
(2.340, 2.215]	75.58 ± 0.09	(2.247, 2.122]	76.39 ± 0.07
(2.305, 2.180]	75.64 ± 0.09	(2.217, 2.092]	76.72 ± 0.07
(2.271, 2.146]	75.75 ± 0.09	(2.187, 2.062]	77.06 ± 0.07
(2.236, 2.111]	75.87 ± 0.09	(2.157, 2.032]	77.43 ± 0.08
(2.202, 2.077]	76.02 ± 0.09	(2.127, 2.002]	77.77 ± 0.07
(2.167, 2.042]	76.21 ± 0.09	(2.097, 1.972]	78.09 ± 0.08
(2.133, 2.008]	76.40 ± 0.09	(2.066, 1.941]	78.35 ± 0.08
(2.098, 1.973]	76.61 ± 0.09	(2.036, 1.911]	78.55 ± 0.08
(2.064, 1.939]	76.83 ± 0.10	(2.006, 1.881]	78.71 ± 0.08
(2.029, 1.904]	77.07 ± 0.10	(1.976, 1.851]	78.86 ± 0.08
(1.995, 1.870]	77.35 ± 0.11	(1.946, 1.821]	79.07 ± 0.09
(1.960, 1.835]	77.70 ± 0.11	(1.916, 1.791]	79.41 ± 0.09
(1.926, 1.801]	78.21 ± 0.10	(1.885, 1.760]	80.02 ± 0.10
(1.891, 1.766]	78.95 ± 0.10	(1.855, 1.730]	81.09 ± 0.12
(1.857, 1.732]	80.12 ± 0.11	(1.825, 1.700]	82.88 ± 0.15

Table S14. Specific range of change of bond lengths for structural ensembles relative to the respective bond lengths in the transition state structure and the unified zero (take the first point as zero point) total configurational entropy calculated in each structural ensemble for the NgnD-catalyzed Diels–Alder reaction. The mean and standard error were calculated from 20 rounds of BGAN-EPS. The ensembles labeled in red font indicate the eliminated ensembles from entropy profiling because the number of samples is below 25 %.

[4+2]-adduct		[6+4]-adduct	
**Bond Range (Å)**	$-TS\ (kcal / mol)$	Bond Range (Å)	$-TS\ (kcal / mol)$
(0.000, -0.125]	0.00 ± 0.10	(0.000, -0.125]	0.00 ± 0.07
(-0.034, -0.159]	-0.49 ± 0.09	(-0.030, -0.155]	-0.25 ± 0.07
(-0.069, -0.194]	-0.82 ± 0.09	(-0.060, -0.185]	-0.44 ± 0.07
(-0.103, -0.228]	-1.03 ± 0.09	(-0.091, -0.216]	-0.57 ± 0.07
(-0.138, -0.263]	-1.18 ± 0.09	(-0.121, -0.246]	-0.63 ± 0.07
(-0.172, -0.297]	-1.26 ± 0.09	(-0.151, -0.276]	-0.63 ± 0.07
(-0.207, -0.332]	-1.29 ± 0.09	(-0.181, -0.306]	-0.58 ± 0.07
(-0.241, -0.366]	-1.27 ± 0.09	(-0.211, -0.336]	-0.49 ± 0.07
(-0.276, -0.401]	-1.20 ± 0.09	(-0.241, -0.366]	-0.38 ± 0.07
(-0.310, -0.435]	-1.09 ± 0.09	(-0.272, -0.397]	-0.27 ± 0.07
(-0.345, -0.470]	-0.99 ± 0.09	(-0.302, -0.427]	-0.15 ± 0.07
(-0.379, -0.504]	-0.90 ± 0.09	(-0.332, -0.457]	0.01 ± 0.07
(-0.414, -0.539]	-0.83 ± 0.09	(-0.362, -0.487]	0.18 ± 0.07
(-0.448, -0.573]	-0.81 ± 0.09	(-0.392, -0.517]	0.38 ± 0.07
(-0.483, -0.608]	-0.76 ± 0.09	(-0.422, -0.547]	0.63 ± 0.07
(-0.517, -0.642]	-0.71 ± 0.09	(-0.453, -0.578]	0.92 ± 0.07
(-0.552, -0.677]	-0.65 ± 0.09	(-0.483, -0.608]	1.25 ± 0.07
(-0.586, -0.711]	-0.54 ± 0.09	(-0.513, -0.638]	1.59 ± 0.07
(-0.621, -0.746]	-0.41 ± 0.09	(-0.543, -0.668]	1.95 ± 0.08
(-0.655, -0.780]	-0.26 ± 0.09	(-0.573, -0.698]	2.29 ± 0.07
(-0.690, -0.815]	-0.07 ± 0.09	(-0.603, -0.728]	2.61 ± 0.08
(-0.724, -0.849]	0.12 ± 0.09	(-0.634, -0.759]	2.87 ± 0.08
(-0.759, -0.884]	0.33 ± 0.09	(-0.664, -0.789]	3.08 ± 0.08
(-0.793, -0.918]	0.55 ± 0.10	(-0.694, -0.819]	3.24 ± 0.08
(-0.828, -0.953]	0.78 ± 0.10	(-0.724, -0.849]	3.39 ± 0.08
(-0.862, -0.987]	1.06 ± 0.11	(-0.754, -0.879]	3.59 ± 0.09
(-0.897, -1.022]	1.42 ± 0.11	(-0.784, -0.909]	3.93 ± 0.09
(-0.931, -1.056]	1.92 ± 0.10	(-0.815, -0.940]	4.54 ± 0.10
(-0.966, -1.091]	2.67 ± 0.10	(-0.845, -0.970]	5.62 ± 0.12
(-1.000, -1.125]	3.84 ± 0.11	(-0.875, -1.000]	7.41 ± 0.15

Table S15. Specific bond range for structural ensembles and the initial total configurational entropy calculated in each structural ensemble for the SpnF-catalyzed Diels–Alder reaction. The mean and standard error were calculated from 20 rounds of BGAN-EPS. The ensembles labeled in red font indicate the eliminated ensembles from entropy profiling because the number of samples is below 25 %.

[4+2]-adduct		[6+4]-adduct	
**Bond Range (Å)**	$-TS\ (kcal\ /\ mol)$	**Bond Range (Å)**	$-TS\ (kcal\ /\ mol)$
(3.056, 2.928]	94.38 ± 0.12	(2.978, 2.850]	98.70 ± 0.11
(3.016, 2.889]	93.93 ± 0.12	(2.938, 2.811]	98.52 ± 0.10
(2.977, 2.849]	93.59 ± 0.12	(2.899, 2.771]	98.60 ± 0.10
(2.937, 2.809]	93.37 ± 0.12	(2.859, 2.731]	98.91 ± 0.10
(2.897, 2.770]	93.20 ± 0.12	(2.819, 2.692]	99.44 ± 0.10
(2.858, 2.730]	93.10 ± 0.12	(2.780, 2.652]	100.02 ± 0.10
(2.818, 2.690]	93.06 ± 0.12	(2.740, 2.612]	100.58 ± 0.10
(2.778, 2.651]	93.03 ± 0.12	(2.700, 2.573]	100.94 ± 0.10
(2.739, 2.611]	93.08 ± 0.12	(2.661, 2.533]	101.12 ± 0.11
(2.699, 2.571]	93.19 ± 0.12	(2.621, 2.493]	101.02 ± 0.12
(2.659, 2.532]	93.40 ± 0.12	(2.581, 2.454]	100.69 ± 0.12
(2.620, 2.492]	93.57 ± 0.12	(2.542, 2.414]	100.25 ± 0.12
(2.580, 2.452]	93.69 ± 0.12	(2.502, 2.374]	99.99 ± 0.12
(2.540, 2.413]	93.73 ± 0.12	(2.462, 2.335]	99.91 ± 0.12
(2.501, 2.373]	93.75 ± 0.12	(2.423, 2.295]	100.07 ± 0.11
(2.461, 2.333]	93.86 ± 0.11	(2.383, 2.255]	100.32 ± 0.10
(2.421, 2.294]	94.02 ± 0.11	(2.343, 2.216]	100.57 ± 0.11
(2.382, 2.254]	94.26 ± 0.11	(2.304, 2.176]	100.75 ± 0.11
(2.342, 2.214]	94.52 ± 0.11	(2.264, 2.136]	100.94 ± 0.12
(2.302, 2.175]	94.78 ± 0.11	(2.224, 2.097]	101.15 ± 0.13
(2.263, 2.135]	95.05 ± 0.10	(2.185, 2.057]	101.38 ± 0.13
(2.223, 2.095]	95.38 ± 0.10	(2.145, 2.017]	101.67 ± 0.14
(2.183, 2.056]	95.74 ± 0.10	(2.105, 1.978]	102.02 ± 0.13
(2.144, 2.016]	96.17 ± 0.11	(2.066, 1.938]	102.52 ± 0.11
(2.104, 1.976]	96.68 ± 0.11	(2.026, 1.898]	103.21 ± 0.11
(2.064, 1.937]	97.21 ± 0.12	(1.986, 1.859]	104.31 ± 0.14
(2.025, 1.897]	97.81 ± 0.13	(1.947, 1.819]	106.26 ± 0.17
(1.985, 1.857]	98.60 ± 0.14	(1.907, 1.779]	109.64 ± 0.23
(1.945, 1.818]	99.77 ± 0.16	(1.867, 1.740]	115.37 ± 0.34
(1.906, 1.778]	101.58 ± 0.18	(1.828, 1.700]	125.76 ± 0.58

Table S16. Specific range of change of bond lengths for structural ensembles relative to the respective bond lengths in the transition state structure and the unified zero (take the first point as zero point) total configurational entropy calculated in each structural ensemble for the SpnF-catalyzed Diels–Alder reaction. The mean and standard error were calculated from 20 rounds of BGAN-EPS. The ensembles labeled in red font indicate the eliminated ensembles from entropy profiling because the number of samples is below 25 %.

[4+2]-adduct		[6+4]-adduct	
**Bond Range (Å)**	$-TS\ (kcal/mol)$	**Bond Range (Å)**	$-TS\ (kcal/mol)$
(0.000, -0.128]	0.00 ± 0.12	(0.000, -0.128]	0.00 ± 0.11
(-0.040, -0.167]	-0.45 ± 0.12	(-0.040, -0.167]	-0.18 ± 0.10
(-0.079, -0.207]	-0.79 ± 0.12	(-0.079, -0.207]	-0.10 ± 0.10
(-0.119, -0.247]	-1.01 ± 0.12	(-0.119, -0.247]	0.21 ± 0.10
(-0.159, -0.286]	-1.18 ± 0.12	(-0.159, -0.286]	0.74 ± 0.10
(-0.198, -0.326]	-1.28 ± 0.12	(-0.198, -0.326]	1.32 ± 0.10
(-0.238, -0.366]	-1.32 ± 0.12	(-0.238, -0.366]	1.88 ± 0.10
(-0.278, -0.405]	-1.35 ± 0.12	(-0.278, -0.405]	2.24 ± 0.10
(-0.317, -0.445]	-1.30 ± 0.12	(-0.317, -0.445]	2.42 ± 0.11
(-0.357, -0.485]	-1.19 ± 0.12	(-0.357, -0.485]	2.32 ± 0.12
(-0.397, -0.524]	-0.98 ± 0.12	(-0.397, -0.524]	1.99 ± 0.12
(-0.436, -0.564]	-0.80 ± 0.12	(-0.436, -0.564]	1.55 ± 0.12
(-0.476, -0.604]	-0.69 ± 0.12	(-0.476, -0.604]	1.29 ± 0.12
(-0.516, -0.643]	-0.65 ± 0.12	(-0.516, -0.643]	1.21 ± 0.12
(-0.555, -0.683]	-0.63 ± 0.12	(-0.555, -0.683]	1.37 ± 0.11
(-0.595, -0.723]	-0.52 ± 0.11	(-0.595, -0.723]	1.62 ± 0.10
(-0.635, -0.762]	-0.36 ± 0.11	(-0.635, -0.762]	1.87 ± 0.11
(-0.674, -0.802]	-0.12 ± 0.11	(-0.674, -0.802]	2.05 ± 0.11
(-0.714, -0.842]	0.14 ± 0.11	(-0.714, -0.842]	2.24 ± 0.12
(-0.754, -0.881]	0.40 ± 0.11	(-0.754, -0.881]	2.45 ± 0.13
(-0.793, -0.921]	0.67 ± 0.10	(-0.793, -0.921]	2.68 ± 0.13
(-0.833, -0.961]	1.00 ± 0.10	(-0.833, -0.961]	2.97 ± 0.14
(-0.873, -1.000]	1.36 ± 0.10	(-0.873, -1.000]	3.32 ± 0.13
(-0.912, -1.040]	1.79 ± 0.11	(-0.912, -1.040]	3.82 ± 0.11
(-0.952, -1.080]	2.30 ± 0.11	(-0.952, -1.080]	4.51 ± 0.11
(-0.992, -1.119]	2.83 ± 0.12	(-0.992, -1.119]	5.61 ± 0.14
(-1.031, -1.159]	3.43 ± 0.13	(-1.031, -1.159]	7.56 ± 0.17
(-1.071, -1.199]	4.22 ± 0.14	(-1.071, -1.199]	10.94 ± 0.23
(-1.111, -1.238]	5.39 ± 0.16	(-1.111, -1.238]	16.67 ± 0.34
(-1.150, -1.278]	7.20 ± 0.18	(-1.150, -1.278]	27.06 ± 0.58

Table S17. Specific bond range for structural ensembles and the unified zero (take the first point as zero point) electronic energy (mean and standard error) calculated in each structural ensemble for the diene/triene cycloaddition. 1961 trajectories were used for each adduct.

**[4+2]-adduct**		**[6+4]-adduct**	
**Bond Range (Å)**	$E\ (kcal\ /\ mol)$	**Bond Range (Å)**	$E\ (kcal\ /\ mol)$
(2.790, 2.658]	0.00 ± 0.07	(3.009, 2.877]	0.00 ± 0.09
(2.658, 2.526]	-2.90 ± 0.07	(2.877, 2.745]	-1.97 ± 0.07
(2.526, 2.394]	-6.05 ± 0.08	(2.745, 2.613]	-4.43 ± 0.07
(2.394, 2.262]	-8.91 ± 0.09	(2.613, 2.481]	-7.61 ± 0.08
(2.262, 2.130]	-11.70 ± 0.10	(2.481, 2.349]	-10.35 ± 0.09
(2.130, 1.998]	-14.74 ± 0.11	(2.349, 2.217]	-12.88 ± 0.09
(1.998, 1.866]	-19.07 ± 0.12	(2.217, 2.085]	-15.21 ± 0.10
(1.866, 1.734]	-23.50 ± 0.13	(2.085, 1.953]	-18.63 ± 0.12
(1.734, 1.602]	-27.97 ± 0.13	(1.953, 1.821]	-22.97 ± 0.13
		(1.821, 1.689]	-28.24 ± 0.14

Table S18. Specific bond range for structural ensembles and the unified zero (take the first point as zero point) electronic energy (mean and standard error) calculated in each structural ensemble for the tethered-diene/triene cycloaddition. 102 trajectories were used for each adduct.

**[4+2]-adduct**		**[6+4]-adduct**	
**Bond Range (Å)**	$E\ (kcal\ /\ mol)$	**Bond Range (Å)**	$E\ (kcal\ /\ mol)$
(2.824, 2.695]	0.00 ± 0.62	(2.742, 2.613]	0.00 ± 0.52
(2.695, 2.565]	-7.02 ± 0.61	(2.613, 2.483]	-6.87 ± 0.56
(2.565, 2.436]	-9.50 ± 0.49	(2.483, 2.354]	-11.11 ± 0.49
(2.436, 2.306]	-13.61 ± 0.52	(2.354, 2.224]	-13.72 ± 0.52
(2.306, 2.177]	-16.42 ± 0.49	(2.224, 2.095]	-16.21 ± 0.57
(2.177, 2.048]	-17.44 ± 0.50	(2.095, 1.966]	-20.12 ± 0.64
(2.048, 1.918]	-20.10 ± 0.56	(1.966, 1.836]	-24.32 ± 0.70
(1.918, 1.789]	-23.59 ± 0.62	(1.836, 1.707]	-29.70 ± 0.73

Table S19. Specific bond range for structural ensembles and the unified zero (take the first point as zero point) electronic energy (mean and standard error) calculated in each structural ensemble for the NgnD-catalyzed Diels–Alder reaction. 117 trajectories were used for each adduct.

**[4+2]-adduct**		**[6+4]-adduct**	
**Bond Range (Å)**	$E\ (kcal\ /\ mol)$	**Bond Range (Å)**	$E\ (kcal\ /\ mol)$
(2.857, 2.732]	0.00 ± 0.53	(2.700, 2.575]	0.00 ± 0.47
(2.732, 2.607]	-3.51 ± 0.48	(2.575, 2.450]	-3.78 ± 0.48
(2.607, 2.482]	-5.56 ± 0.50	(2.450, 2.325]	-4.89 ± 0.48
(2.482, 2.357]	-6.76 ± 0.47	(2.325, 2.200]	-7.23 ± 0.54
(2.357, 2.232]	-7.87 ± 0.43	(2.200, 2.075]	-8.93 ± 0.61
(2.232, 2.107]	-8.01 ± 0.43	(2.075, 1.950]	-10.70 ± 0.65

(2.107, 1.982]	-9.76 ± 0.53	(1.950, 1.825]	-13.36 ± 0.72
(1.982, 1.857]	-11.66 ± 0.63	(1.825, 1.700]	-16.15 ± 0.78
(1.857, 1.732]	-14.22 ± 0.74		

Table S20. Specific bond range for structural ensembles and the unified zero (take the first point as zero point) electronic energy (mean and standard error) calculated in each structural ensemble for the SpnF-catalyzed Diels–Alder reaction. 121 trajectories were used for each adduct.

**[4+2]-adduct**		**[6+4]-adduct**	
**Bond Range (Å)**	$E\ (kcal\ /\ mol)$	**Bond Range (Å)**	$E\ (kcal\ /\ mol)$
(3.056, 2.928]	0.00 ± 0.31	(2.978, 2.850]	0.00 ± 0.32
(2.928, 2.800]	-0.78 ± 0.26	(2.850, 2.722]	-1.39 ± 0.28
(2.800, 2.673]	-1.12 ± 0.25	(2.722, 2.595]	-1.70 ± 0.29
(2.673, 2.545]	-1.22 ± 0.25	(2.595, 2.467]	-2.25 ± 0.33
(2.545, 2.417]	-1.87 ± 0.32	(2.467, 2.339]	-2.27 ± 0.37
(2.417, 2.289]	-1.77 ± 0.36	(2.339, 2.211]	-2.82 ± 0.40
(2.289, 2.161]	-2.53 ± 0.44	(2.211, 2.083]	-3.12 ± 0.50
(2.161, 2.034]	-4.28 ± 0.53	(2.083, 1.956]	-4.73 ± 0.59
(2.034, 1.906]	-7.67 ± 0.64	(1.956, 1.828]	-7.37 ± 0.70
(1.906, 1.778]	-9.92 ± 0.71	(1.828, 1.700]	-7.85 ± 0.74
